# Impact of STROBE Statement Publication on Quality of Observational Study Reporting: Interrupted Time Series *versus* Before-After Analysis

**DOI:** 10.1371/journal.pone.0064733

**Published:** 2013-08-26

**Authors:** Sylvie Bastuji-Garin, Emilie Sbidian, Caroline Gaudy-Marqueste, Emilie Ferrat, Jean-Claude Roujeau, Marie-Aleth Richard, Florence Canoui-Poitrine, on behalf of the European Dermatology Network (EDEN)

**Affiliations:** 1 Université Paris Est Créteil (UPEC), LIC EA4393 (Laboratoire d'Investigation Clinique), Créteil, France; 2 AP-HP, Hôpital Henri-Mondor, Department of Clinical Research and Public Health, Créteil, France; 3 AP-HP, Hôpital Henri-Mondor, Unité de Recherche Clinique (URC), Créteil, France; 4 AP-HP, Hôpital Henri-Mondor, Department of Dermatology, Créteil, France; 5 AP-HM, Timone University, Department of Dermatology, Marseille, France; 6 Université Paris Est Créteil (UPEC), Faculté de Medecine, Department of General Practice, Créteil, France; CUNY, United States of America

## Abstract

**Background:**

In uncontrolled before-after studies, CONSORT was shown to improve the reporting of randomised trials. Before-after studies ignore underlying secular trends and may overestimate the impact of interventions. Our aim was to assess the impact of the 2007 STROBE statement publication on the quality of observational study reporting, using both uncontrolled before-after analyses and interrupted time series.

**Methods:**

For this quasi-experimental study, original articles reporting cohort, case-control, and cross-sectional studies published between 2004 and 2010 in the four dermatological journals having the highest 5-year impact factors (≥4) were selected. We compared the proportions of STROBE items (STROBE score) adequately reported in each article during three periods, **two pre STROBE period (2004–2005 and 2006–2007) and one post STROBE period (2008–2010).** Segmented regression analysis of interrupted time series was also performed.

**Results:**

Of the 456 included articles, 187 (41%) reported cohort studies, 166 (36.4%) cross-sectional studies, and 103 (22.6%) case-control studies. The median STROBE score was 57% (range, 18%–98%). Before-after analysis evidenced significant STROBE score increases between the two pre-STROBE periods and between the earliest pre-STROBE period and the post-STROBE period (median score_2004–05_ 48% *versus* median score_2008–10_ 58%, *p*<0.001) but not between the immediate pre-STROBE period and the post-STROBE period (median score_2006–07_ 58% *versus* median score_2008–10_ 58%, *p* = 0.42). **In the pre STROBE period**, the six-monthly mean STROBE score increased significantly, by 1.19% per six-month period (absolute increase 95%CI, 0.26% to 2.11%, *p* = 0.016). By segmented analysis, no significant changes in STROBE score trends occurred (−0.40%; 95%CI, −2.20 to 1.41; *p* = 0.64) **in the post STROBE statement publication**.

**Interpretation:**

The quality of reports increased over time but was not affected by STROBE. Our findings raise concerns about the relevance of uncontrolled before-after analysis for estimating the impact of guidelines.

## Introduction

The randomised controlled design is the reference standard for evaluating the efficacy of new treatments but cannot answer all important questions about a given intervention. Observational studies may be better able to detect rare or delayed adverse effects of treatments and to reflect outcomes obtained in everyday practice [1]. However, the reporting of observational research may be insufficiently accurate or clear to enable assessments of the strengths and weaknesses of the available evidence [2], [3]. To improve the reporting of observational cohort, case-control, and cross-sectional studies, a group of experts developed a checklist of 22-items, which was published in 2007 as the STrengthening the Reporting of OBservational Studies in Epidemiology (STROBE) statement [4], [5], [6], [7], [8], [9], [10]. A few studies used STROBE to assess the quality of observational study reporting [11], [12]; however, the impact of STROBE on the quality of observational study reporting has never been assessed **excepted 2 randomized studies assessing the impact of adding the STROBE checklist to conventional review on manuscript quality** [13], [14]. According to uncontrolled before-after studies, the 1996 CONsolidated Standards of Reporting Trials (CONSORT) statement improved the reporting of randomised trials [15], [16], [17]. However, the uncontrolled before-after design fails to take underlying secular trends into account, which may result in overestimation of the impact of interventions [18], [19]. Furthermore, reporting quality is generally assessed in leading generalist medical journals with very high impact factors (IF), whereas most studies are published in specialist journals.

The goals of this study were to test the hypotheses that the quality of observational study reporting improved over time and that the generally used uncontrolled before-after design was inadequate for assessing whether STROBE statement publication affected this improvement. We assessed the quality of observational study reporting between 2004 and 2010 in the four dermatological journals with the highest 5-year IFs.

## Methods

For this quasi-experimental study, we selected the four dermatology journals with the highest 5-year IFs in the 2010 Journal Citation Report, namely, the *Journal of Investigative Dermatology* (IF, 5.76), the *British Journal of Dermatology* (IF, 4.24), the *Journal of the American Academy of Dermatology* (IF, 4.16), and the *Archives of Dermatology* (IF, 3.98). *Pigment Cell and Melanoma Research* (IF, 4.64) was not included because this journal publishes nearly only experimental studies.

### Data selection

We selected all articles published between January 2004 and December 2010 that reported cohort, case-control, or cross-sectional studies. We did not include non-original studies, experimental and basic science studies, meta-analyses, letters, or studies in categories having their own reporting guidelines, namely, diagnostic and genetic studies (STARD and STREGA, respectively).

**Figure 1:**
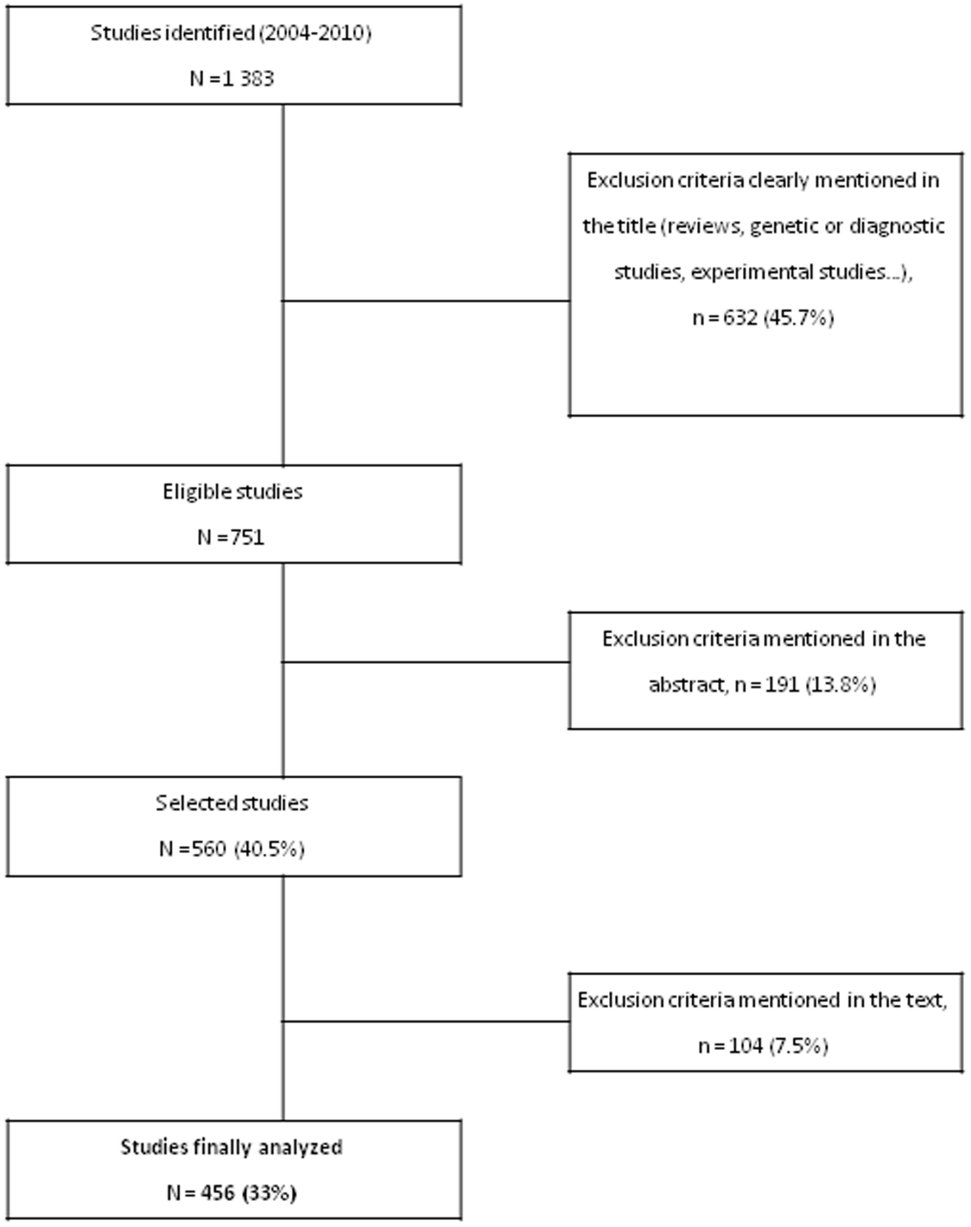
Flow diagram.

To identify eligible studies, we conducted a PubMed search of Medline and we manually searched all issues of each journal published during the study period. The indexing terms used for the electronic search were ((“Case-Control Studies”[Mesh] OR “Cohort Studies”[Mesh] OR “Cross-Sectional Studies”[Mesh]) AND (“the British Journal of Dermatology” [journal] OR “the Journal of the American Academy of Dermatology” [journal] OR “the Archives of Dermatology” [journal] OR “the Journal of Investigative Dermatology” [journal]) NOT “Randomized Controlled Trial”[Publication Type]) with limits: “humans, only items with abstracts, English”. The titles and abstracts were screened by two of us (SBG and ES) working independently of each other and resolving disagreements by consensus, which led to the selection of 560 articles (Figure 1). The names and affiliations of the authors and the dates of article acceptance and publication were masked to minimise **evaluation** bias.

### Data abstraction

To standardise the data abstraction process and to determine whether further clarification of STROBE item scoring was needed, all of us performed a pilot experiment consisting in abstracting data from 25 articles. All articles were then allocated at random to pairs of investigators; each pair was composed of a physician specialised in clinical epidemiology (PhD) and a dermatologist. Discrepancies were reviewed within the pairs and resolved by consensus if possible; if not, one of us (SBG) served as the arbitrator. To avoid potential bias due to working in pairs and to ensure consistency in the review process throughout the study, a permutation scheme was used to modify the pairs. **Permutation scheme permitted to disseminate learning and to improve pair performance.**

The investigators abstracted the 22 items of the STROBE checklist by answering 57 questions (online supplement) adapted from those used by Langan et al. [11] Four response options were available for each of the 57 questions: ‘yes’, ‘in part or unclear’, ‘no’, and ‘not applicable’. The acceptance year and publication year of each article were extracted by one of us (SBG), who was blinded to the checklist answers. Data were collected using an electronic case-report form established specifically for the study (CleanWEB^©^, *Telemedicine Technologies S.A.–2007*).

### Outcome measure

The primary outcome was the STROBE score, defined as the number of the 22 STROBE items adequately reported divided by the number of applicable items, expressed as a percentage. The 13 STROBE items with several questions (2 to 15 questions per item, online supplement) were considered adequately reported when at least 50% of their questions had ‘yes’ answers (after exclusion of the ‘not applicable’ components) [11].

This study did not require approval by an ethics committee, since it concerned publications and not individuals.

### Data analysis

Quantitative variables are reported as median (interquartile range) and qualitative variables as number (percentage).

#### Uncontrolled before-after analysis

We used the Mann-Whitney test with Bonferroni's correction to compare STROBE scores of articles published in 2004–5 (early pre-STROBE period) and 2008–10 (post-STROBE period). Then, we compared the early and immediate pre-STROBE periods (2004–5 *versus* 2006–7) and the immediate pre-STROBE and post-STROBE periods (2006–7 *versus* 2008–10). **We assessed the** ***a posteriori* power**.

#### Interrupted time series analysis

We used a segmented linear regression model to determine the impact of STROBE over time [18], [19], [20]. We considered two periods, the pre-STROBE period (from the first semester of 2004 to the second semester of 2007) and the post-STROBE period (from the first semester of 2008 to the second semester of 2010). Because we hypothesised that STROBE statement dissemination increased gradually over time, we did not consider a dissemination segment for the principal analysis.

The dependent variable was the six-month STROBE score mean. A period of six months was chosen to obtain at least 30 observations per point. The independent variable was the semester and year of publication.

The segmented regression model included an intercept (α1), a baseline trend (β1), and a change of trend after STROBE publication (β2). The level and trend of the pre-STROBE segment (2004–2007) served as the control for the post-STROBE segment (2008–2010). We estimated the difference between pre-STROBE and post-STROBE slopes and the six-monthly mean STROBE effect after STROBE publication. Independence of residuals was tested using the autocorrelation function and the Durbin-Watson test. Similar analyses stratified by journal were performed.

#### Sensitivity analyses

Similar analyses were also performed using two sensitivity-STROBE scores. For the first score, “partly” answers were analysed as “yes” answers. The second score considered the proportion of the 57 STROBE-derived questions that were adequately answered. For this score, 1 was assigned to ‘yes’ answers and 0.5 to ‘in part or unclear’ answers to obtain a sum that was then divided by the number of applicable questions. Similar analyses were performed with the post-STROBE period restricted to 2009–10 and with three periods, a pre-STROBE period (2004–2007), a dissemination period (2008), and a post-STROBE period (2009–2010) (interrupted time series). **Last, similar analyses were performed using non pooled data (with the dependant variable being the STROBE score per article).**

All tests were two-tailed, and *p* values <0.05 were considered significant.

Data were analysed using STATA v11.0 (College Station, TX, USA) and SAS v9.3 (SAS Institute, Cary, NC, USA) software.

## Results

Of the 560 initially selected articles, 104 (18.6%) were excluded after reviewing the full publication because they were not relevant to the study (86 case-series, 11 interventional studies, 5 genetic studies, and 2 diagnostic studies). Figure 1 shows the flow diagram. The list of articles is provided in the online supplement.

The remaining 456 articles reported 187 (41%) cohort studies, 166 (36.4%) cross-sectional studies, and 103 (22.6%) case-control studies. The median number of articles per year was 63 (range 47 to 91). The median STROBE score was 57% (range 18 to 98%). **Details regarding the reporting of the different items of STROBE are available on** **Table 1** **.** Table 2 displays the median STROBE score values over time. There were no missing data.

**Table 1:** Proportion of adequate reporting of the 22 items of the STROBE statement in the 456 articles analyzed.

	Item No	Recommendation	N	(%)
**Title and abstract**	1	(*a*) Indicate the study's design with a commonly used term in the title or the abstract	296	(64.9)
		(*b*) Provide in the abstract an informative and balanced summary of what was done and what was found		
**Introduction**				
Background/rationale	2	Explain the scientific background and rationale for the investigation being reported	402	(88.2)
Objectives	3	State specific objectives, including any prespecified hypotheses	396	(86.8)
**Methods**				
Study design	4	Present key elements of study design early in the paper	210	(46.1)
Setting	5	Describe the setting, locations, and relevant dates, including periods of recruitment, exposure, follow-up, and data collection	352	(77.2)
Participants	6	(*a*) *Cohort study*—Give the eligibility criteria, and the sources and methods of selection of participants. Describe methods of follow-up *Case-control study*—Give the eligibility criteria, and the sources and methods of case ascertainment and control selection. Give the rationale for the choice of cases and controls *Cross-sectional study*—Give the eligibility criteria, and the sources and methods of selection of participants	348	(76.3)
		(*b*) *Cohort study*—For matched studies, give matching criteria and number of exposed and unexposed *Case-control study*—For matched studies, give matching criteria and the number of controls per case		
Variables	7	Clearly define all outcomes, exposures, predictors, potential confounders, and effect modifiers. Give diagnostic criteria, if applicable	227	(49.9)
Data sources/measurement	8*	For each variable of interest, give sources of data and details of methods of assessment (measurement). Describe comparability of assessment methods if there is more than one group	339	(74.3)
Bias	9	Describe any efforts to address potential sources of bias	124	(27.2)
Study size	10	Explain how the study size was arrived at	19	(4.5)
Quantitative variables	11	Explain how quantitative variables were handled in the analyses. If applicable, describe which groupings were chosen and why	174	(42.1)
Statistical methods	12	(*a*) Describe all statistical methods, including those used to control for confounding	73	(16)
		(*b*) Describe any methods used to examine subgroups and interactions		
		(*c*) Explain how missing data were addressed		
		(*d*) *Cohort study*—If applicable, explain how loss to follow-up was addressed *Case-control study*—If applicable, explain how matching of cases and controls was addressed *Cross-sectional study*—If applicable, describe analytical methods taking account of sampling strategy		
		(*e*) Describe any sensitivity analyses		
**Results**				
Participants	13*	(a) Report numbers of individuals at each stage of study—eg numbers potentially eligible, examined for eligibility, confirmed eligible, included in the study, completing follow-up, and analysed	124	(33.2)
		(b) Give reasons for non-participation at each stage		
		(c) Consider use of a flow diagram		
Descriptive data	14*	(a) Give characteristics of study participants (eg demographic, clinical, social) and information on exposures and potential confounders	274	(60.8)
		(b) Indicate number of participants with missing data for each variable of interest		
		(c) *Cohort study*—Summarise follow-up time (eg, average and total amount)		
Outcome data	15*	*Cohort study*—Report numbers of outcome events or summary measures over time	145	(92.4)
		*Case-control study*—Report numbers in each exposure category, or summary measures of exposure		
		*Cross-sectional study*—Report numbers of outcome events or summary measures		
Main results	16	(*a*) Give unadjusted estimates and, if applicable, confounder-adjusted estimates and their precision (eg, 95% confidence interval). Make clear which confounders were adjusted for and why they were included	284	(62.8)
		(*b*) Report category boundaries when continuous variables were categorized		
		(*c*) If relevant, consider translating estimates of relative risk into absolute risk for a meaningful time period		
Other analyses	17	Report other analyses done—eg analyses of subgroups and interactions, and sensitivity analyses	170	(38.5)
**Discussion**				
Key results	18	Summarise key results with reference to study objectives	326	(71.5)
Limitations	19	Discuss limitations of the study, taking into account sources of potential bias or imprecision. Discuss both direction and magnitude of any potential bias	208	(45.6)
Interpretation	20	Give a cautious overall interpretation of results considering objectives, limitations, multiplicity of analyses, results from similar studies, and other relevant evidence	210	(46)
Generalisability	21	Discuss the generalisability (external validity) of the study results	127	(28.2)
**Other information**				
Funding	22	Give the source of funding and the role of the funders for the present study and, if applicable, for the original study on which the present article is based	278	(61)

* Give information separately for cases and controls in case-control studies and, if applicable, for exposed and unexposed groups in cohort and cross-sectional studies.

**Table 2:** Quality of the reports of observational studies as assessed using the STROBE score over time.

	Pre-STROBE publication				Post-STROBE publication			*p* value for the two-by-two comparisons*		
Years	2004	2005	2006	2007	2008	2009	2010	Pre-1 *vs.* Post	Pre-1 *vs.* Pre-2	Pre 2 *vs.* Post
	(n = 47)	(n = 63)	(n = 63)	(n = 62)	(n = 69)	(n = 91)	(n = 61)			
STROBE Score, Median (IQR)	47 (40–63)	48 (38–61)	58 (47–67)	60 (46–71)	57 (41–67)	59 (48–73)	59 (48–76)			
Periods	Early pre-STROBE 2004–5 (pre-1)		Immediate pre-STROBE 2006–7 (pre-2)		Post-STROBE 2008–10 (post)					
STROBE score, Median (IQR)	48 (39–61)		58 (46–68)		58 (46–73)			<0.001	<0.001	0.42

IQR, interquartile range.

*p* value by the Mann-Whitney test; *p* values ≤0.016 were considered significant according to Bonferroni's correction.

The STROBE items adequately reported in less than 50% of articles were sample size estimation (5% of adequate reporting), statistical methods (16%), description of efforts to limit potential sources of bias (27%), discussion of external validity (28%), number of participants at each stage (33%), statistical treatment of quantitative variables (42%), and discussion of limitations (46%).

### Before-after analysis

The STROBE score increased significantly from the early pre-STROBE period to the post-STROBE period (median score_2004–05_ 48% *versus* median score_2008–10_ 58%, *p*<0.001) and between the two pre-STROBE periods (Table 2). Conversely, STROBE scores did not change significantly between the immediate pre-STROBE period (2006–7) and the post-STROBE period (median score_2006–07_ 58% *versus* median score_2008–10_ 58%, *p* = 0.42).

Similar results were obtained with the sensitivity-STROBE scores, and with the post-STROBE period restricted to 2009–10 (data not shown).

### Time series analysis

**Figure 2:**
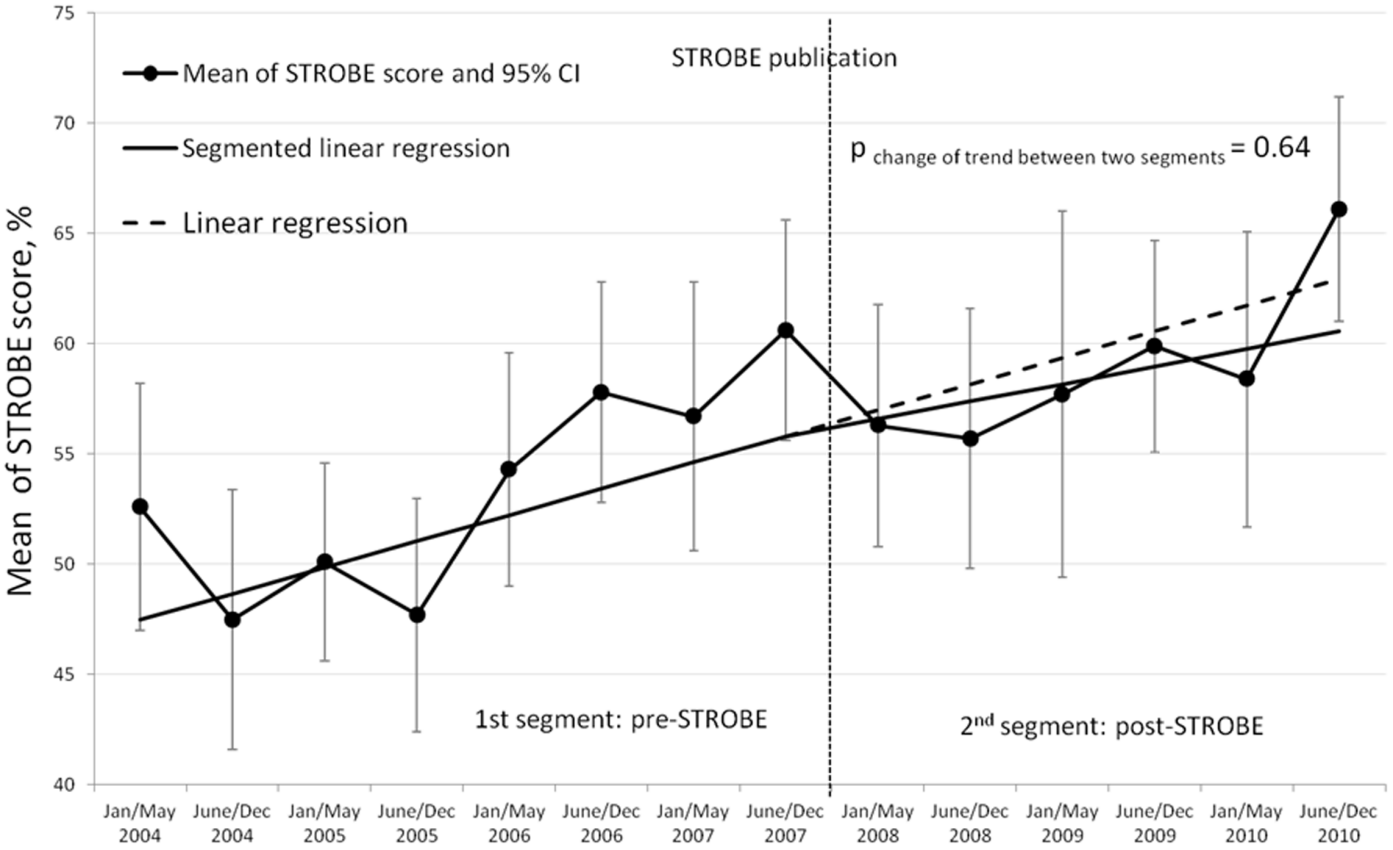
Time series of six-monthly mean STROBE scores and values predicted from the segmented and simple linear regression models.

**In the pre STROBE** statement publication period, the mean STROBE score increased significantly, by 1.19% per six-month period (95% confidence interval [95%CI] of the absolute increase, 0.26% to 2.11%, *p* = 0.016) (Figure 2). This trend did not change significantly after publication of the STROBE statement (absolute change, −0.40%; 95%CI, −2.20 to 1.41; *p* = 0.64).

Table 3 reports the baseline trend and change in trend after STROBE statement publication using the full linear segmented model and the most parsimonious model consisting in simple linear regression after elimination of non-significant terms (change between the before and after periods). Our final model was neither corrected for seasonal variations (not applicable) nor adjusted for autocorrelation (residuals were independent, normally distributed, with mean zero and constant variance). Finally, the six-monthly mean STROBE score increased by 1.01% (absolute increase 95%CI, 0.58% to 1.44%, *p*<0.001).

**Table 3:** Parameter estimates from the full and most parsimonious final linear regression models predicting the mean six-monthly STROBE score per article.

	Estimate coefficient (standard deviation)	*p* value
**Full model (segmented linear regression)**		
1st segment (pre-STROBE, 1^st^ half of 2004 to 2^nd^ half of 2007)		
Intercept α1	47.5 (2.31)	<0.001
Baseline trend β1	1.19 (0.42)	0.016
2^nd^ segment (post-STROBE, 1^st^ half of 2008 to 2^nd^ half of 2010)		
Trend change β2	−0.40 (0.82)	0.64
**Final model (Simple linear regression)** †		
Intercept	48.2 (1.69)	<0.001
Trend change β1	1.01 (0.20)	<0.001

† The final model (i.e., the most parsimonious model) included all the terms significant in the segmented model.

In stratified analyses, the baseline STROBE score differed across journals, but the trends were almost similar across the four journals (Figure S1 online supplement).

Results were very similar with the sensitivity-STROBE scores or with a dissemination period (data not shown). **In the non pooled analyses, the STROBE score per article increased significantly, by 2.1% per year (95%CI of the absolute increase, 1.3% to 2.8%,** ***p*<10^−4^).**

#### *A posteriori* power

**Based on the sample size including in the 3 periods 110, 125 and 221 articles respectively and a mean STROBE score of 50/100 with a standard deviation of 15, we were able to detect a 5 points increase with a power of 80%.”**

## Interpretation

We found that reporting was inadequate in a large proportion of articles published from 2004 to 2010, the median STROBE score being 57%. Reporting rates were lowest for sample size estimation, description of statistical methods and of efforts to limit potential sources of bias, discussion of external validity, and discussion of limitations. By uncontrolled before-after analysis, the STROBE score increased significantly between the early pre-STROBE period (2004–5) and 2008–10 but not between the immediate pre-STROBE period (2006–7) and 2008–10. Interrupted time series analysis showed a significant STROBE score increase over time that was not influenced by the publication of STROBE.

The few studies assessing the quality of observational study reporting, with the STROBE statement as a reference, identified a number of deficiencies consistent with our findings, including marked inadequacies in reporting the management of missing data [11], [12], [21], [22], confounding [21], [22], and sample size [11], [21], [22]. The global STROBE score for 2006–2007 of 58% was close to the median number of reported items per article found by Langan et al. (59%, 55%, and 55% for cohort, cross-sectional, and case-control studies, respectively) in five dermatology journals (2005–2007) [11]. Interestingly, somewhat higher global STROBE scores were reported for studies in leading generalist journals (69% in 2010) [22], in accordance with the lower and delayed compliance with CONSORT in specialty publications compared to generalist journals such as the *New England Journal of Medicine* or *The Lancet* [23]. Although the general applicability of our findings from dermatology journals may be debatable, we believe that assessing reporting quality in specialist journals is crucial, since these journals account for the majority of studies that are published and read by specialists on a regular basis. None of the studies assessing quality of observational study reporting [11], [12], [21], [22] evaluated the impact of STROBE statement publication. Several studies suggested that using the CONSORT statement might improve the reporting of randomised controlled trials [15], [16], [17], [24]. However, all these studies used the uncontrolled before-after design. Previous evidence suggests that uncontrolled before-after analyses comparing two time periods may overestimate the effects of interventions designed to improve quality [18]. In keeping with this possibility, our before-after analysis showed a significant improvement between two time points in the **pre and post STROBE statement publication periods**. Interrupted time series analysis is a strong quasi-experimental method for distinguishing the baseline trend from the effect of interventions in longitudinal studies [19], [20]. A well-designed time series analysis increases the confidence with which the estimated effect can be attributed to the intervention, although it does not separate the intervention-related effect from the potential effects of other events occurring at the same time [18].

**Our study did not support evidence of a significant impact of STROBE statement publication during the study period**. It may be related to two main factors. First, STROBE was published at a time of continuous improvements in reporting quality in medical journals, extending across all study designs, which may have masked additional subtle benefits related to STROBE. Second, our research covers only the first three years after STROBE publication. It would be of interest to evaluate subsequent trends, particularly given the recent endorsement of the STROBE statement by two of the four journals included in our study (*British Journal of Dermatology* and *Journal of the American Academy of Dermatology*). Endorsement of a reporting guideline by a journal may have a greater impact on reporting quality in that journal than publication of the guideline. However, during our study period, none of the four journals had endorsed STROBE, in keeping with most other medical journals. We aimed to analyse penetration of STROBE and not its endorsement by journals. Moreover, in a comparison of the quality of reporting of randomised controlled trials in four journals, of which three required the use of CONSORT from 1996 onwards (*JAMA, British Medical Journal, The Lancet*) and one did not (*New England Journal of Medicine*), a before-after analysis indicated an improvement in quality between 1994 and 1998 in all four journals [15].

### Limitations

We did not analyse agreement between the pairs of reviewers, but the permutation scheme used to modify the pairs limited potential bias related to working in pairs while ensuring consistency in the review process throughout the study. We used a global score for each article to provide a measure of overall reporting. In choosing this method, we do not suggest that all items are of equal importance. We built two sensitivity-STROBE scores; the consistency of the sensitivity analysis results with the main analysis supports the robustness of our findings.

The factor with the strongest influence on the quality of time series analysis is the number of data points collected **in the pre-intervention period (estimation of trend) and in the post intervention period (estimation of the intervention effect)** [19], [20]. We considered only eight data points in the pre-STROBE period, but this number is higher than the three data points recommended by the Cochrane Effective Practice and Organisation of Care group to obtain a stable underlying secular trend [25].

## Conclusion

This study highlights continuing deficiencies in the reporting of observational studies in dermatology journals despite improvements over time (2004–2010). Our results suggest that publication of the STROBE statement may have failed to significantly influence the quality of observational study reporting during the first three years. Moreover, we illustrated that the uncontrolled before-after design may produce inaccurate results regarding the impact of study reporting guidelines. The impact of reporting guidelines should be assessed using the adequate methods currently used for assessing medical practice guidelines or public health interventions.

## Supporting Information

Figure S1 **Time series of annual mean STROBE scores and values predicted from simple linear regression models stratified by journal.** The y axis shows the annual mean STROBE score by journal and the x axis the year.
(TIF)

Text S1 **Indexing terms used for the electronic search, list of articles included in the study.**
(DOC)
