# Supplementary figures and images for: Impact of STROBE Statement Publication on Quality of Observational Study Reporting: Interrupted Time Series versus Before-After Analysis
Source: PLoS One. 2013 Aug 26;8(8):e64733. doi: 10.1371/journal.pone.0064733 (PMC3753332; doi:10.1371/journal.pone.0064733)

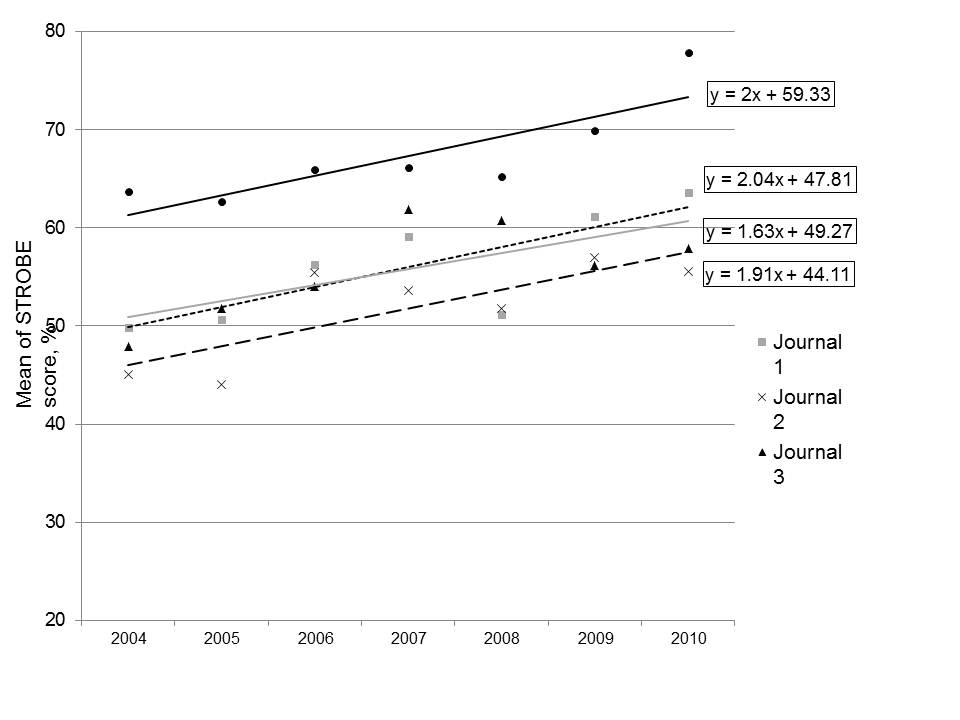

Supplement: Figure S1 — Time series of annual mean STROBE scores and values predicted from simple linear regression models stratified by journal. The y axis shows the annual mean STROBE score by journal and the x axis the year. (TIF) [file pone.0064733.s001.tif]
