# Supplementary material for: Impact of STROBE Statement Publication on Quality of Observational Study Reporting: Interrupted Time Series versus Before-After Analysis
Source: PLoS One. 2013 Aug 26;8(8):e64733. doi: 10.1371/journal.pone.0064733 (PMC3753332; doi:10.1371/journal.pone.0064733)
Supplement: Text S1 — Indexing terms used for the electronic search, list of articles included in the study. (DOC) [file pone.0064733.s002.doc]

*Indexing terms used for the electronic search*

(("Case-Control Studies"[Mesh] OR "Cohort Studies"[Mesh] OR "Cross-Sectional Studies"[Mesh]) AND ("the British Journal of Dermatology" [journal] OR “the Journal of the American Academy of Dermatology” [journal] OR “the Archives of Dermatology” [journal] OR “ the Journal of Investigative Dermatology” [journal]) NOT "Randomized Controlled Trial "[Publication Type]) with limits: “humans, only items with abstracts, English”.

*List of articles included in the study*

1. Aalborg J, Morelli JG, Byers TE, Mokrohisky ST, Crane LA. Effect of hair color and sun sensitivity on nevus counts in white children in Colorado. J Am Acad Dermatol. 2010 Sep;63(3):430-9.
2. Abasq C, Mouquet H, Gilbert D, Tron F, Grassi V, Musette P, Joly P. ELISA testing of anti-desmoglein 1 and 3 antibodies in the management of pemphigus. Arch Dermatol. 2009 May;145(5):529-35.
3. Abdel Fattah NS, Darwish YW. Is there a role for insulin resistance in nonobese patients with idiopathic hirsutism? Br J Dermatol. 2009 May;160(5):1011-5.
4. Abeni D, Frontani M, Sampogna F, Sera F, Bolli S, Corona R, Baliva G, Russo G. Circulating CD8+ lymphocytes, white blood cells, and survival in patients with mycosis fungoides. Br J Dermatol. 2005 Aug; 153(2):324-30.
5. Adebamowo CA, Spiegelman D, Berkey CS, Danby FW, Rockett HH, Colditz GA, Willett WC, Holmes MD. Milk consumption and acne in teenaged boys. J Am Acad Dermatol. 2008 May;58(5):787-93.
6. Adebamowo CA, Spiegelman D, Danby FW, Frazier AL, Willett WC, Holmes MD. High school dietary dairy intake and teenage acne. J Am Acad Dermatol. 2005 Feb;52(2):207-14.
7. Alivernini S, De Santis M, Tolusso B, Mannocci A, Bosello SL, Peluso G, Pinnelli M, D'Antona G, LaTorre G, Ferraccioli G. Skin ulcers in systemic sclerosis: determinants of presence and predictive factors of healing. J Am Acad Dermatol. 2009 Mar;60(3):426-35.
8. Alpsoy E, Donmez L, Onder M, Gunasti S, Usta A, Karincaoglu Y, Kandi B, Buyukkara S, Keseroglu O, Uzun S, Tursen U, Seyhan M, Akman A. Clinical features and natural course of Behçet's disease in 661 cases: a multicentre study. Br J Dermatol. 2007 Nov;157(5):901-6.
9. Ambros-Rudolph CM, Hofmann-Wellenhof R, Richtig E, Müller-Fürstner M, Soyer HP, Kerl H. Malignant melanoma in marathon runners. Arch Dermatol. 2006 Nov;142(11):1471-4.
10. Amici JM, Rogues AM, Lasheras A, Gachie JP, Guillot P, Beylot C, Thomas L, Taïeb A. A prospective study of the incidence of complications associated with dermatological surgery. Br J Dermatol. 2005 Nov;153(5):967-71.
11. Annesi-Maesano I, Beyer A, Marmouz F, Mathelier-Fusade P, Vervloet D, Bauchau V. Do patients with skin allergies have higher levels of anxiety than patients with allergic respiratory diseases? Results of a large-scale cross-sectional study in a French population. Br J Dermatol. 2006 Jun;154(6):1128-36.
12. Antoniou C, Stefanaki I, Stratigos A, Moustou E, Vergou T, Stavropoulos P, Avgerinou G, Rigopoulos D, Katsambas AD. Infliximab for the treatment of psoriasis in Greece: 4 years of clinical experience at a single centre. Br J Dermatol. 2010 May;162(5):1117-23.
13. Applebaum KM, Nelson HH, Zens MS, Stukel TA, Spencer SK, Karagas MR. Oral contraceptives: a risk factor for squamous cell carcinoma? J Invest Dermatol. 2009 Dec;129(12):2760-5. Epub 2009 Jun 25. PubMed PMID: 19554020; PubMed Central PMCID: PMC2928055.
14. Arana A, Wentworth CE, Fernández-Vidaurre C, Schlienger RG, Conde E, Arellano FM. Incidence of cancer in the general population and in patients with or without atopic dermatitis in the U.K. Br J Dermatol. 2010 Nov;163(5):1036-43. doi: 10.1111/j.1365-2133.2010.09887.x.
15. Arellano FM, Wentworth CE, Arana A, Fernández C, Paul CF. Risk of lymphoma following exposure to calcineurin inhibitors and topical steroids in patients with atopic dermatitis. J Invest Dermatol. 2007 Apr;127(4):808-16.
16. Argenziano G, Kittler H, Ferrara G, Rubegni P, Malvehy J, Puig S, Cowell L, Stanganelli I, De Giorgi V, Thomas L, Bahadoran P, Menzies SW, Piccolo D, Marghoob AA, Zalaudek I. Slow-growing melanoma: a dermoscopy follow-up study. Br J Dermatol. 2010 Feb 1;162(2):267-73.
17. Arias-Santiago S, Gutiérrez-Salmerón MT, Buendía-Eisman A, Girón-Prieto MS, Naranjo-Sintes R. Hypertension and aldosterone levels in women with early-onset androgenetic alopecia. Br J Dermatol. 2010 Apr;162(4):786-9.
18. Arias-Santiago S, Gutiérrez-Salmerón MT, Castellote-Caballero L, Naranjo-Sintes R. Elevated aldosterone levels in patients with androgenetic alopecia. Br J Dermatol. 2009 Nov;161(5):1196-8.
19. Arias-Santiago S, Gutiérrez-Salmerón MT, Castellote-Caballero L, Buendía-Eisman A, Naranjo-Sintes R. Androgenetic alopecia and cardiovascular risk factors in men and women: a comparative study. J Am Acad Dermatol. 2010 Sep;63(3):420-9.
20. Asgari MM, Chren MM, Warton EM, Friedman GD, White E. Association between nonsteroidal anti-inflammatory drug use and cutaneous squamous cell carcinoma. Arch Dermatol. 2010 Apr;146(4):388-95.
21. Asgari MM, Maruti SS, Kushi LH, White E. A cohort study of vitamin D intake and melanoma risk. J Invest Dermatol. 2009 Jul;129(7):1675-80. Epub 2009 Feb 5.
22. Asgari MM, Tang J, Epstein EH Jr, Chren MM, Warton EM, Quesenberry CP Jr, Go AS, Friedman GD. Statin use and risk of basal cell carcinoma. J Am Acad Dermatol. 2009 Jul;61(1):66-72.
23. Asgari MM, Tang J, Warton ME, Chren MM, Quesenberry CP Jr, Bikle D, Horst RL, Orentreich N, Vogelman JH, Friedman GD. Association of prediagnostic serum vitamin D levels with the development of basal cell carcinoma. J Invest Dermatol. 2010 May;130(5):1438-43.
24. Athavale P, Shum KW, Chen Y, Agius R, Cherry N, Gawkrodger DJ; EPIDERM. Occupational dermatitis related to chromium and cobalt: experience of dermatologists (EPIDERM) and occupational physicians (OPRA) in the U.K. over an 11-year period (1993-2004). Br J Dermatol. 2007 Sep;157(3):518-22.
25. Autier J, Picard-Dahan C, Marinho E, Grossin M, Yeni P, Leport C, Vildé JL, Crickx B, Descamps V. Docetaxel in anthracycline-pretreated AIDS-related Kaposi's sarcoma: a retrospective study. Br J Dermatol. 2005 May;152(5):1026-9.
26. Azoulay L, Oraichi D, Bérard A. Isotretinoin therapy and the incidence of acne relapse: a nested case-control study. Br J Dermatol. 2007 Dec;157(6):1240-8.
27. Azzarello LM, Jacobsen PB. Factors influencing participation in cutaneous screening among individuals with a family history of melanoma. J Am Acad Dermatol. 2007 Mar;56(3):398-406.
28. Baade PD, English DR, Youl PH, McPherson M, Elwood JM, Aitken JF. The relationship between melanoma thickness and time to diagnosis in a large population-based study. Arch Dermatol. 2006 Nov;142(11):1422-7.
29. Baade PD, Youl PH, Janda M, Whiteman DC, Del Mar CB, Aitken JF. Factors associated with the number of lesions excised for each skin cancer: a study of primary care physicians in Queensland, Australia. Arch Dermatol. 2008 Nov;144(11):1468-76.
30. Baccarelli A, Pesatori AC, Consonni D, Mocarelli P, Patterson DG Jr, Caporaso NE, Bertazzi PA, Landi MT. Health status and plasma dioxin levels in chloracne cases 20 years after the Seveso, Italy accident. Br J Dermatol. 2005 Mar;152(3):459-65.
31. Bahrami S, Malone JC, Webb KG, Callen JP. Tissue eosinophilia as an indicator of drug-induced cutaneous small-vessel vasculitis. Arch Dermatol. 2006 Feb;142(2):155-61.
32. Bamford JT, Gessert CE, Renier CM, Jackson MM, Laabs SB, Dahl MV, Rogers RS 3rd. Childhood stye and adult rosacea. J Am Acad Dermatol. 2006 Dec;55(6):951-5.
33. Bangsgaard N, Engkilde K, Thyssen JP, Linneberg A, Nielsen NH, Menné T, Skov L, Johansen JD. Inverse relationship between contact allergy and psoriasis: results from a patient- and a population-based study. Br J Dermatol. 2009 Nov;161(5):1119-23.
34. Banky JP, Kelly JW, English DR, Yeatman JM, Dowling JP. Incidence of new and changed nevi and melanomas detected using baseline images and dermoscopy in patients at high risk for melanoma. Arch Dermatol. 2005 Aug;141(8):998-1006.
35. Barahmani N, Schabath MB, Duvic M; National Alopecia Areata Registry. History of atopy or autoimmunity increases risk of alopecia areata. J Am Acad Dermatol. 2009 Oct;61(4):581-91.
36. Barlow JO, Zalla MJ, Kyle A, DiCaudo DJ, Lim KK, Yiannias JA. Treatment of basal cell carcinoma with curettage alone. J Am Acad Dermatol. 2006 Jun;54(6):1039-45.
37. Baron SE, Goodwin RG, Nicolau N, Blackford S, Goulden V. Use of complementary medicine among outpatients with dermatologic conditions within Yorkshire and South Wales, United Kingdom. J Am Acad Dermatol. 2005 Apr;52(4):589-94.
38. Baron SE, Morris PK, Dye L, Fielding D, Goulden V. The effect of dermatology consultations in secondary care on treatment outcome and quality of life in new adult patients with atopic dermatitis. Br J Dermatol. 2006 May;154(5):942-9.
39. Barzilai DA, Cooper KD, Neuhauser D, Rimm AA, Cooper GS. Geographic and patient variation in receipt of surveillance procedures after local excision of cutaneous melanoma. J Invest Dermatol. 2004 Feb;122(2):246-55.
40. Battistella M, Fraitag S, Teillac DH, Brousse N, de Prost Y, Bodemer C. Neonatal and early infantile cutaneous langerhans cell histiocytosis: comparison of self-regressive and non-self-regressive forms. Arch Dermatol. 2010 Feb;146(2):149-56.
41. Bendewald MJ, Wetter DA, Li X, Davis MD. Incidence of dermatomyositis and clinically amyopathic dermatomyositis: a population-based study in Olmsted County, Minnesota. Arch Dermatol. 2010 Jan;146(1):26-30.
42. Ben-Gashir MA, Seed PT, Hay RJ. Predictors of atopic dermatitis severity over time. J Am Acad Dermatol. 2004 Mar;50(3):349-56.
43. Benner MF, Willemze R. Applicability and prognostic value of the new TNM classification system in 135 patients with primary cutaneous anaplastic large cell lymphoma. Arch Dermatol. 2009 Dec;145(12):1399-404.
44. Bernard P, Reguiai Z, Tancrède-Bohin E, Cordel N, Plantin P, Pauwels C, Vaillant L, Grange F, Richard-Lallemand MA, Sassolas B, Roujeau JC, Lok C, Picard-Dahan C, Chosidow O, Vitry F, Joly P. Risk factors for relapse in patients with bullous pemphigoid in clinical remission: a multicenter, prospective, cohort study. Arch Dermatol. 2009 May;145(5):537-42.
45. Bett BJ. Large or multiple congenital melanocytic nevi: occurrence of neurocutaneous melanocytosis in 1008 persons. J Am Acad Dermatol. 2006 May;54(5):767-77.
46. Biagini Myers JM, Wang N, LeMasters GK, Bernstein DI, Epstein TG, Lindsey MA, Ericksen MB, Chakraborty R, Ryan PH, Villareal MS, Burkle JW, Lockey JE, Reponen T, Khurana Hershey GK. Genetic and environmental risk factors for childhood eczema development and allergic sensitization in the CCAAPS cohort. J Invest Dermatol. 2010 Feb;130(2):430-7.
47. Binder B, Lackner HK, Salmhofer W, Kroemer S, Custovic J, Hofmann-Wellenhof R. Association between superficial vein thrombosis and deep vein thrombosis of the lower extremities. Arch Dermatol. 2009 Jul;145(7):753-7.
48. Bingham LG, Noble JW, Davis MD. Wet dressings used with topical corticosteroids for pruritic dermatoses: A retrospective study. J Am Acad Dermatol. 2009 May;60(5):792-800.
49. Birlea SA, Fain PR, Spritz RA. A Romanian population isolate with high frequency of vitiligo and associated autoimmune diseases. Arch Dermatol. 2008 Mar;144(3):310-6.
50. Birnie AJ, Thomas KS, Varma S. Should eye protection be worn during dermatological surgery: prospective observational study. Br J Dermatol. 2007 Jun;156(6):1258-62
51. Blake PW, Bradford PT, Devesa SS, Toro JR. Cutaneous appendageal carcinoma incidence and survival patterns in the United States: a population-based study. Arch Dermatol. 2010 Jun;146(6):625-32.
52. Boeckler P, Cosnes A, Francès C, Hedelin G, Lipsker D. Association of cigarette smoking but not alcohol consumption with cutaneous lupus erythematosus. Arch Dermatol. 2009 Sep;145(9):1012-6.
53. Bormann G, Marsch WC, Haerting J, Helmbold P. Concomitant traumas influence prognosis in melanomas of the nail apparatus. Br J Dermatol. 2006 Jul;155(1):76-80.
54. Bouwes Bavinck JN, Euvrard S, Naldi L, Nindl I, Proby CM, Neale R, Abeni D, Tessari GP, Feltkamp MC, Claudy A, Stockfleth E, Harwood CA; EPI-HPV-UV-CA group. Keratotic skin lesions and other risk factors are associated with skin cancer in organ-transplant recipients: a case-control study in The Netherlands, United Kingdom, Germany, France, and Italy. J Invest Dermatol. 2007 Jul;127(7):1647-56.
55. Bowe WP, Leyden JJ, Crerand CE, Sarwer DB, Margolis DJ. Body dysmorphic disorder symptoms among patients with acne vulgaris. J Am Acad Dermatol. 2007 Aug;57(2):222-30.
56. Boztepe G, Akinci H, Sahin S, Karaduman A, Evans SE, Erkin G, Atakan N, Akan T, Kölemen F. In search of an optimum dose escalation for narrowband UVB phototherapy: is it time to quit 20% increments? J Am Acad Dermatol. 2006 Aug;55(2):269-71.
57. Bradford PT, Freedman DM, Goldstein AM, Tucker MA. Increased risk of second primary cancers after a diagnosis of melanoma. Arch Dermatol. 2010 Mar;146(3):265-72.
58. Brauchli YB, Jick SS, Curtin F, Meier CR. Association between beta-blockers, other antihypertensive drugs and psoriasis: population-based case-control study. Br J Dermatol. 2008 Jun;158(6):1299-307.
59. Brauchli YB, Jick SS, Curtin F, Meier CR. Association between use of thiazolidinediones or other oral antidiabetics and psoriasis: A population based case-control study. J Am Acad Dermatol. 2008 Mar;58(3):421-9.
60. Brauchli YB, Jick SS, Meier CR. Psoriasis and the risk of incident diabetes mellitus: a population-based study. Br J Dermatol. 2008 Dec;159(6):1331-7.
61. Brauchli YB, Jick SS, Miret M, Meier CR. Psoriasis and risk of incident cancer: an inception cohort study with a nested case-control analysis. J Invest Dermatol. 2009 Nov;129(11):2604-12.
62. Brauchli YB, Jick SS, Miret M, Meier CR. Psoriasis and risk of incident myocardial infarction, stroke or transient ischaemic attack: an inception cohort study with a nested case-control analysis. Br J Dermatol. 2009 May;160(5):1048-56.
63. Bremmer SF, Hanifin JM, Simpson EL. Clinical detection of ichthyosis vulgaris in an atopic dermatitis clinic: implications for allergic respiratory disease and prognosis. J Am Acad Dermatol. 2008 Jul;59(1):72-8.
64. Brenninkmeijer EE, Spuls PI, Legierse CM, Lindeboom R, Smitt JH, Bos JD. Clinical differences between atopic and atopiform dermatitis. J Am Acad Dermatol. 2008 Mar;58(3):407-14.
65. Brewer JD, Colegio OR, Phillips PK, Roenigk RK, Jacobs MA, Van de Beek D, Dierkhising RA, Kremers WK, McGregor CG, Otley CC. Incidence of and risk factors for skin cancer after heart transplant. Arch Dermatol. 2009 Dec;145(12):1391-6.
66. Brooks K, Brooks D, Dajani Z, Swetter SM, Powers E, Pagoto S, Geller AC. Use of artificial tanning products among young adults. J Am Acad Dermatol. 2006 Jun;54(6):1060-6.
67. Brunasso AM, Massone C. Thrombocytopenia associated with the use of anti-tumor necrosis factor-alpha agents for psoriasis. J Am Acad Dermatol. 2009 May;60(5):781-5.
68. Bub JL, Berg D, Slee A, Odland PB. Management of lentigo maligna and lentigo maligna melanoma with staged excision: a 5-year follow-up. Arch Dermatol. 2004 May;140(5):552-8.
69. Buckley DA, Basketter DA, Smith Pease CK, Rycroft RJ, White IR, McFadden JP. Simultaneous sensitivity to fragrances. Br J Dermatol 2006 May;154(5):885-8
70. Buldanlioglu S, Turkmen S, Ayabakan HB, Yenice N, Vardar M, Dogan S, Mercan E. Nitric oxide, lipid peroxidation and antioxidant defence system in patients with active or inactive Behçet's disease. Br J Dermatol. 2005 Sep;153(3):526-30.
71. Butler GJ, Neale R, Green AC, Pandeya N, Whiteman DC. Nonsteroidal anti-inflammatory drugs and the risk of actinic keratoses and squamous cell cancers of the skin. J Am Acad Dermatol. 2005 Dec;53(6):966-72.
72. Campisi G, Di Fede O, Craxi A, Di Stefano R, Margiotta V. Oral lichen planus, hepatitis C virus, and HIV: no association in a cohort study from an area of high hepatitis C virus endemicity. J Am Acad Dermatol. 2004 Sep;51(3):364-70.
73. Cappel M, Mauger D, Thiboutot D. Correlation between serum levels of insulin-like growth factor 1, dehydroepiandrosterone sulfate, and dihydrotestosterone and acne lesion counts in adult women. Arch Dermatol. 2005 Mar;141(3):333-8.
74. Carli P, De Giorgi V, Crocetti E, Mannone F, Massi D, Chiarugi A, Giannotti B. Improvement of malignant/benign ratio in excised melanocytic lesions in the 'dermoscopy era': a retrospective study 1997-2001. Br J Dermatol. 2004 Apr;150(4):687-92.
75. Carli P, Ghigliotti G, Gnone M, Chiarugi A, Crocetti E, Astorino S, Berti UA, Broganelli P, Carcaterra A, Corradin MT, Pellacani G, Piccolo D, Risulo M, Stanganelli I, De Giorgi V. Baseline factors influencing decisions on digital follow-up of melanocytic lesions in daily practice: an Italian multicenter survey. J Am Acad Dermatol. 2006 Aug;55(2):256-62.
76. Carlsen BC, Andersen KE, Menné T, Johansen JD. Sites of dermatitis in a patch test population: hand dermatitis is associated with polysensitization. Br J Dermatol. 2009 Oct;161(4):808-13.
77. Cartwright T, Endean N, Porter A. Illness perceptions, coping and quality of life in patients with alopecia. Br J Dermatol. 2009 May;160(5):1034-9
78. Cham PM, Chen SC, Grill JP, Warshaw EM. Validity of self-reported nail counts in patients with onychomycosis: A retrospective pilot analysis. J Am Acad Dermatol. 2008 Jan;58(1):136-41.
79. Chan YC, Giam YC. A retrospective cohort study of Southeast Asian patients with large congenital melanocytic nevi and the risk of melanoma development. J Am Acad Dermatol. 2006 May;54(5):778-82.
80. Cheetham TC, Wagner RA, Chiu G, Day JM, Yoshinaga MA, Wong L. A risk management program aimed at preventing fetal exposure to isotretinoin: retrospective cohort study. J Am Acad Dermatol. 2006 Sep;55(3):442-8.
81. Chen CL, Kuppermann M, Caughey AB, Zane LT. A community-based study of acne-related health preferences in adolescents. Arch Dermatol. 2008 Aug;144(8):988-94.
82. Chen T, Bertenthal D, Sahay A, Sen S, Chren MM. Predictors of skin-related quality of life after treatment of cutaneous basal cell carcinoma and squamous cell carcinoma. Arch Dermatol. 2007 Nov;143(11):1386-92.
83. Chen YJ, Wu CY, Shen JL, Chu SY, Chen CK, Chang YT, Chen CM. Psoriasis independently associated with hyperleptinemia contributing to metabolic syndrome. Arch Dermatol. 2008 Dec;144(12):1571-5.
84. Chi CC, Wang SH, Charles-Holmes R, Ambros-Rudolph C, Powell J, Jenkins R, Black M, Wojnarowska F. Pemphigoid gestationis: early onset and blister formation are associated with adverse pregnancy outcomes. Br J Dermatol. 2009 Jun;160(6):1222-8.
85. Chia CY, Lane W, Chibnall J, Allen A, Siegfried E. Isotretinoin therapy and mood changes in adolescents with moderate to severe acne: a cohort study. Arch Dermatol. 2005 May;141(5):557-60.
86. Chiu LS, Chow VC, Ling JM, Hon KL. Staphylococcus aureus carriage in the anterior nares of close contacts of patients with atopic dermatitis. Arch Dermatol. 2010 Jul;146(7):748-52.
87. Chiu LS, Ho MS, Hsu LY, Tang MB. Prevalence and molecular characteristics of Staphylococcus aureus isolates colonizing patients with atopic dermatitis and their close contacts in Singapore. Br J Dermatol. 2009 May;160(5):965-71.
88. Choi K, Lazovich D, Southwell B, Forster J, Rolnick SJ, Jackson J. Prevalence and characteristics of indoor tanning use among men and women in the United States. Arch Dermatol. 2010 Dec;146(12):1356-61.
89. Chren MM, Sahay AP, Bertenthal DS, Sen S, Landefeld CS. Quality-of-life outcomes of treatments for cutaneous basal cell carcinoma and squamous cell carcinoma. J Invest Dermatol. 2007 Jun;127(6):1351-7.
90. Cicek D, Kandi B, Berilgen MS, Bulut S, Tekatas A, Dertlioglu SB, Ozel S, Saral Y. Does autonomic dysfunction play a role in atopic dermatitis. Br J Dermatol 2008 Sep;159(4):834-8
91. Clark FL, Sahay A, Bertenthal D, Maddock L, Lindquist K, Grekin R, Chren MM. Variation in care for recurrent nonmelanoma skin cancer in a university-based practice and a veterans affairs clinic. Arch Dermatol. 2008 Sep;144(9):1148-52.
92. Clayton TH, Wilkinson SM, Rawcliffe C, Pollock B, Clark SM. Allergic contact dermatitis in children: should pattern of dermatitis determine referral? A retrospective study of 500 children tested between 1995 and 2004 in one U.K. centre. Br J Dermatol. 2006 Jan;154(1):114-7.
93. Cohen AD, Ofek-Shlomai A, Vardy DA, Weiner Z, Shvartzman P. Depression in dermatological patients identified by the Mini International Neuropsychiatric Interview questionnaire. J Am Acad Dermatol. 2006 Jan;54(1):94-9.
94. Cokkinides VE, Bandi P, Weinstock MA, Ward E. Use of sunless tanning products among US adolescents aged 11 to 18 years. Arch Dermatol. 2010 Sep;146(9):987-92.
95. Colbert RL, Allen DM, Eastwood D, Fairley JA. Mortality rate of bullous pemphigoid in a US medical center. J Invest Dermatol. 2004 May;122(5):1091-5
96. Cooper SM, Ali I, Baldo M, Wojnarowska F. The association of lichen sclerosus and erosive lichen planus of the vulva with autoimmune disease: a case-control study. Arch Dermatol. 2008 Nov;144(11):1432-5.
97. Cooper SM, Gao XH, Powell JJ, Wojnarowska F. Does treatment of vulvar lichen sclerosus influence its prognosis? Arch Dermatol. 2004 Jun;140(6):702-6.
98. Cottoni F, Masala MV, Pattaro C, Pirodda C, Montesu MA, Satta R, Cerimele D, de Marco R. Classic Kaposi sarcoma in northern Sardinia: a prospective epidemiologic overview (1977-2003) correlated with malaria prevalence (1934). J Am Acad Dermatol. 2006 Dec;55(6):990-5.
99. Couppié P, Clyti E, Sobesky M, Bissuel F, Del Giudice P, Sainte-Marie D, Dedet JP, Carme B, Pradinaud R. Comparative study of cutaneous leishmaniasis in human immunodeficiency virus (HIV)-infected patients and non-HIV-infected patients in French Guiana. Br J Dermatol. 2004 Dec;151(6):1165-71.
100. Cox NH. Oedema as a risk factor for multiple episodes of cellulitis/erysipelas of the lower leg: a series with community follow-up. Br J Dermatol. 2006 Nov;155(5):947-50.
101. Crane LA, Mokrohisky ST, Dellavalle RP, Asdigian NL, Aalborg J, Byers TE, Zeng C, Barón AE, Burch JM, Morelli JG. Melanocytic nevus development in Colorad children born in 1998: a longitudinal study. Arch Dermatol. 2009 Feb;145(2):148-56.
102. Cvetkovski RS, Zachariae R, Jensen H, Olsen J, Johansen JD, Agner T. Prognosis of occupational hand eczema: a follow-up study. Arch Dermatol. 2006 Mar;142(3):305-11.
103. Dalgard F, Gieler U, Holm JØ, Bjertness E, Hauser S. Self-esteem and body satisfaction among late adolescents with acne: results from a population survey. J Am Acad Dermatol. 2008 Nov;59(5):746-51.
104. Dalgard F, Svensson A, Holm JØ, Sundby J. Self-reported skin morbidity in Oslo. Associations with sociodemographic factors among adults in a cross-sectional study. Br J Dermatol. 2004 Aug;151(2):452-7.
105. Dalgard F, Svensson A, Sundby J, Dalgard OS. Self-reported skin morbidity and mental health. A population survey among adults in a Norwegian city. Br J Dermatol. 2005 Jul;153(1):145-9.
106. Dalle S, Beylot-Barry M, Bagot M, Lipsker D, Machet L, Joly P, Dompmartin A, d'Incan M, Maubec E, Grange F, Dereure O, Prey S, Barete S, Wetterwald M, Fraitag S, Petrella T. Blastic plasmacytoid dendritic cell neoplasm: is transplantation the treatment of choice? Br J Dermatol. 2010 Jan;162(1):74-9.
107. Damstra RJ, van Steensel MA, Boomsma JH, Nelemans P, Veraart JC. Erysipelas as a sign of subclinical primary lymphoedema: a prospective quantitative scintigraphic study of 40 patients with unilateral erysipelas of the leg. Br J Dermatol. 2008 Jun;158(6):1210-5.
108. Dapprich DC, Weenig RH, Rohlinger AL, Weaver AL, Quan KK, Keeling JH, Walsh JS, Otley CC, Christenson LJ. Outcomes of melanoma in recipients of solid organ transplant. J Am Acad Dermatol. 2008 Sep;59(3):405-17.
109. David M, Tsukrov B, Adler B, Hershko K, Pavlotski F, Rozenman D, Hodak E, Paltiel O. Actinic damage among patients with psoriasis treated by climatotherapy at the Dead Sea. J Am Acad Dermatol. 2005 Mar;52(3 Pt 1):445-50.
110. Davis MD, el-Azhary RA, Farmer SA. Results of patch testing to a corticosteroid series: a retrospective review of 1188 patients during 6 years at Mayo Clinic. J Am Acad Dermatol. 2007 Jun;56(6):921-7.
111. De Giorgi V, Sestini S, Grazzini M, Janowska A, Boddi V, Lotti T. Prevalence and distribution of melanocytic naevi on the scalp: a prospective study. Br J Dermatol. 2010 Feb 1;162(2):345-9.
112. de Jager ME, van de Kerkhof PC, de Jong EM, Seyger MM. A cross-sectional study using the Children's Dermatology Life Quality Index (CDLQI) in childhood psoriasis: negative effect on quality of life and moderate correlation of CDLQI with severity scores. Br J Dermatol. 2010 Nov;163(5):1099-101
113. de Vries E, Louwman M, Bastiaens M, de Gruijl F, Coebergh JW. Rapid and continuous increases in incidence rates of basal cell carcinoma in the southeast Netherlands since 1973. J Invest Dermatol. 2004 Oct;123(4):634-8
114. Debarbieux S, Duru G, Dalle S, Béatrix O, Balme B, Thomas L. Sentinel lymph node biopsy in melanoma: a micromorphometric study relating to prognosis and completion lymph node dissection. Br J Dermatol. 2007 Jul;157(1):58-67.
115. Dellavalle RP, Hester EJ, Stegner DL, Deas AM, Pacheco TR, Mokrohisky S, Morelli JG, Crane LA. Is high mole count a marker of more than melanoma risk? Eczema diagnosis is associated with melanocytic nevi in children. Arch Dermatol. 2004 May;140(5):577-80.
116. Demierre MF, Tien A, Miller D. Health-related quality-of-life assessment in patients with cutaneous T-cell lymphoma. Arch Dermatol. 2005 Mar;141(3):325-30.
117. Derk CT, Huaman G, Jimenez SA. A retrospective randomly selected cohort study of D-penicillamine treatment in rapidly progressive diffuse cutaneous systemic sclerosis of recent onset. Br J Dermatol. 2008 May;158(5):1063-8. Blasdale C, Lawrence CM. Perioperative international normalized ratio level is a poor predictor of postoperative bleeding complications in dermatological surgery patients taking warfarin. Br J Dermatol. 2008 Mar;158(3):522-6.
118. Diepgen TL, Andersen KE, Brandao FM, Bruze M, Bruynzeel DP, Frosch P, Gonçalo M, Goossens A, Le Coz CJ, Rustemeyer T, White IR, Agner T; European Environmental and Contact Dermatitis Research Group. Hand eczema classification: a cross-sectional, multicentre study of the aetiology and morphology of hand eczema. Br J Dermatol. 2009 Feb;160(2):353-8.
119. Dixon AJ, Dixon MP, Dixon JB, Del Mar CB. Prospective study of skin surgery in smokers vs. nonsmokers. Br J Dermatol. 2009 Feb;160(2):365-7.
120. Dobbinson S, Wakefield M, Hill D, Girgis A, Aitken JF, Beckmann K, Reeder AI, Herd N, Fairthorne A, Bowles KA. Prevalence and determinants of Australian adolescents' and adults' weekend sun protection and sunburn, summer 2003-2004. J Am Acad Dermatol. 2008 Oct;59(4):602-14.
121. Dompmartin A, Acher A, Thibon P, Tourbach S, Hermans C, Deneys V, Pocock B, Lequerrec A, Labbé D, Barrellier MT, Vanwijck R, Vikkula M, Boon LM. Association of localized intravascular coagulopathy with venous malformations. Arch Dermatol. 2008 Jul;144(7):873-7.
122. Dreiher J, Shapiro J, Cohen AD. Lichen planus and dyslipidaemia: a case-control study. Br J Dermatol. 2009 Sep;161(3):626-9.
123. Dreiher J, Weitzman D, Cohen AD. Psoriasis and osteoporosis: a sex-specific association? J Invest Dermatol. 2009 Jul;129(7):1643-9.
124. Dupuy A, Schulz T, Chevret S, Agbalika F, Pellet C, Janier M, Dupin N, Vérola O, Calvo F, Lebbé C. Asymmetrical transmission of human herpesvirus 8 among spouses of patients with Kaposi sarcoma. Br J Dermatol. 2009 Mar;160(3):540-5.
125. Duquia RP, Baptista Menezes AM, Reichert FF, de Almeida HL Jr. Prevalence and associated factors with sunscreen use in Southern Brazil: A population-based study. J Am Acad Dermatol. 2007 Jul;57(1):73-80.
126. Durbec F, Vitry F, Granel-Brocard F, Lipsker D, Aubin F, Hédelin G, Dalac S, Truchetet F, Michel C, Batard ML, Domissy-Baury B, Halna JM, Schmutz JL, Delvincourt C, Reuter G, Dalle S, Bernard P, Danzon A, Grange F. The role of circumstances of diagnosis and access to dermatological care in early diagnosis of cutaneous melanoma: a population-based study in France. Arch Dermatol. 2010 Mar;146(3):240-6.
127. El-Gamal H, Bennett RG. Increased breast cancer risk after radiotherapy for acne among women with skin cancer. J Am Acad Dermatol. 2006 Dec;55(6):981-9.
128. Engasser HC, Warshaw EM. Dermatoscopy use by US dermatologists: a cross-sectional survey. J Am Acad Dermatol. 2010 Sep;63(3):412-9, 419.e1-2
129. Evers AW, Verhoeven EW, Kraaimaat FW, de Jong EM, de Brouwer SJ, Schalkwijk J, Sweep FC, van de Kerkhof PC. How stress gets under the skin: cortisol and stress reactivity in psoriasis. Br J Dermatol. 2010 Nov;163(5):986-91. doi: 10.1111/j.1365-2133.2010.09984.x.
130. Fardet L, Cabane J, Lebbé C, Morel P, Flahault A. Incidence and risk factors for corticosteroid-induced lipodystrophy: a prospective study. J Am Acad Dermatol. 2007 Oct;57(4):604-9.
131. Fardet L, Flahault A, Kettaneh A, Tiev KP, Généreau T, Tolédano C, Lebbé C, Cabane J. Corticosteroid-induced clinical adverse events: frequency, risk factors and patient's opinion. Br J Dermatol. 2007 Jul;157(1):142-8.
132. Farhi D, Falissard B, Dupuy A. Global assessment of psoriasis severity and change from photographs: a valid and consistent method. J Invest Dermatol. 2008 Sep;128(9):2198-203.
133. Feramisco JD, Leitenberger JJ, Redfern SI, Bian A, Xie XJ, Resneck JS Jr. A gender gap in the dermatology literature? Cross-sectional analysis of manuscript authorship trends in dermatology journals during 3 decades. J Am Acad Dermatol. 2009 Jan;60(1):63-9.
134. Fine JD, Hall M, Weiner M, Li KP, Suchindran C. The risk of cardiomyopathy in inherited epidermolysis bullosa. Br J Dermatol. 2008 Sep;159(3):677-82.
135. Fine JD, Johnson LB, Weiner M, Li KP, Suchindran C. Epidermolysis bullosa and the risk of life-threatening cancers: the National EB Registry experience, 1986-2006. J Am Acad Dermatol. 2009 Feb;60(2):203-11.
136. Firooz A, Ghandi N, Hallaji Z, Chams-Davatchi C, Valikhani M, Karbakhsh Davari M. Role of thiopurine methyltransferase activity in the safety and efficacy of azathioprine in the treatment of pemphigus vulgaris. Arch Dermatol. 2008 Sep;144(9):1143-7.
137. Fisher NM, Schaffer JV, Berwick M, Bolognia JL. Breslow depth of cutaneous melanoma: impact of factors related to surveillance of the skin, including prior skin biopsies and family history of melanoma. J Am Acad Dermatol. 2005 Sep;53(3):393-406.
138. Florell SR, Meyer LJ, Boucher KM, Porter-Gill PA, Hart M, Erickson J, Cannon-Albright LA, Pershing LK, Harris RM, Samlowski WE, Zone JJ, Leachman SA. Longitudinal assessment of the nevus phenotype in a melanoma kindred. J Invest Dermatol. 2004 Sep;123(3):576-82.
139. Fortes C, Mastroeni S, Leffondré K, Sampogna F, Melchi F, Mazzotti E, Pasquini P, Abeni D. Relationship between smoking and the clinical severity of psoriasis. Arch Dermatol. 2005 Dec;141(12):1580-4.
140. Fortina AB, Piaserico S, Caforio AL, Abeni D, Alaibac M, Angelini A, Iliceto S, Peserico A. Immunosuppressive level and other risk factors for basal cell carcinoma and squamous cell carcinoma in heart transplant recipients. Arch Dermatol. 2004 Sep;140(9):1079-85.
141. Fortune DG, Richards HL, Kirby B, McElhone K, Main CJ, Griffiths CE. Successful treatment of psoriasis improves psoriasis-specific but not more general aspects of patients' well-being. Br J Dermatol. 2004 Dec;151(6):1219-26.
142. Fuhrmann T, Smith N, Tausk F. Use of complementary and alternative medicine among adults with skin disease: updated results from a national survey. J Am Acad Dermatol. 2010 Dec;63(6):1000-5.
143. Lipsker D, Engel F, Cribier B, Velten M, Hedelin G. Trends in melanoma epidemiology suggest three different types of melanoma. Br J Dermatol. 2007 Aug;157(2):338-43.
144. Gaudy-Marqueste C, Madjlessi N, Guillot B, Avril MF, Grob JJ. Risk factors in elderly people for lentigo maligna compared with other melanomas: a double case-control study. Arch Dermatol. 2009 Apr;145(4):418-23.
145. Gelfand JM, Dommasch ED, Shin DB, Azfar RS, Kurd SK, Wang X, Troxel AB. The risk of stroke in patients with psoriasis. J Invest Dermatol. 2009 Oct;129(10):2411-8.
146. Gelfand JM, Shin DB, Neimann AL, Wang X, Margolis DJ, Troxel AB. The risk of lymphoma in patients with psoriasis. J Invest Dermatol. 2006 Oct;126(10):2194-201.
147. Gelfand JM, Troxel AB, Lewis JD, Kurd SK, Shin DB, Wang X, Margolis DJ, Strom BL. The risk of mortality in patients with psoriasis: results from population-based study. Arch Dermatol. 2007 Dec;143(12):1493-9.
148. Gelfand JM, Weinstein R, Porter SB, Neimann AL, Berlin JA, Margolis DJ. Prevalence and treatment of psoriasis in the United Kingdom: a population-based study. Arch Dermatol. 2005 Dec;141(12):1537-41.
149. George ME, Sharma V, Jacobson J, Simon S, Nopper AJ. Adverse effects of systemic glucocorticosteroid therapy in infants with hemangiomas. Arch Dermatol. 2004 Aug;140(8):963-9.
150. Gerami P, Rosen S, Kuzel T, Boone SL, Guitart J. Folliculotropic mycosis fungoides: an aggressive variant of cutaneous T-cell lymphoma. Arch Dermatol. 2008 Jun;144(6):738-46.
151. Gerami P, Wickless SC, Rosen S, Kuzel TM, Ciurea A, Havey J, Guitart J. Applying the new TNM classification system for primary cutaneous lymphomas other than mycosis fungoides and Sézary syndrome in primary cutaneous marginal zone lymphoma. J Am Acad Dermatol. 2008 Aug;59(2):245-54.
152. Gerdes S, Zahl VA, Knopf H, Weichenthal M, Mrowietz U. Comedication related to comorbidities: a study in 1203 hospitalized patients with severe psoriasis. Br J Dermatol. 2008 Nov;159(5):1116-23.
153. Ghodsi SZ, Orawa H, Zouboulis CC. Prevalence, severity, and severity risk factors of acne in high school pupils: a community-based study. J Invest Dermatol. 2009 Sep;129(9):2136-41. Epub 2009 Mar 12. PubMed PMID: 19282841
154. Gholam P, Denk K, Sehr T, Enk A, Hartmann M. Factors influencing pain intensity during topical photodynamic therapy of complete cosmetic units for actinic keratoses. J Am Acad Dermatol. 2010 Aug;63(2):213-8.
155. Gild R, Clay CD, Morey S. Aquagenic wrinkling of the palms in cystic fibrosis and the cystic fibrosis carrier state: a case–control study. Br J Dermatol. 2010 Nov;163(5):1082-4.
156. Gisondi P, Tessari G, Conti A, Piaserico S, Schianchi S, Peserico A, Giannetti A, Girolomoni G. Prevalence of metabolic syndrome in patients with psoriasis: a hospital-based case-control study. Br J Dermatol. 2007 Jul;157(1):68-73.
157. Glatz M, Golestani M, Kerl H, Müllegger RR. Clinical relevance of different IgG and IgM serum antibody responses to Borrelia burgdorferi after antibiotic therapy for erythema migrans: long-term follow-up study of 113 patients. Arch Dermatol. 2006 Jul;142(7):862-8.
158. Grange F, Vitry F, Granel-Brocard F, Lipsker D, Aubin F, Hédelin G, Dalac S, Truchetet F, Michel C, Batard ML, Baury B, Halna JM, Schmutz JL, Delvincourt C, Reuter G, Dalle S, Bernard P, Danzon A. Variations in management of stage I to stage III cutaneous melanoma: a population-based study of clinical practices in France. Arch Dermatol. 2008 May;144(5):629-36.
159. Grob JJ, Revuz J, Ortonne JP, Auquier P, Lorette G. Comparative study of the impact of chronic urticaria, psoriasis and atopic dermatitis on the quality of life. Br J Dermatol. 2005 Feb;152(2):289-95.
160. Gudi VS, White MI, Cruickshank N, Herriot R, Edwards SL, Nimmo F, Ormerod AD. Annual incidence and mortality of bullous pemphigoid in the Grampian Region of North-east Scotland. Br J Dermatol. 2005 Aug; 153(2):424-7.
161. Gueudry J, Roujeau JC, Binaghi M, Soubrane G, Muraine M. Risk factors for the development of ocular complications of Stevens-Johnson syndrome and toxic epidermal necrolysis. Arch Dermatol. 2009 Feb;145(2):157-62.
162. Guttman-Yassky E, Kra-Oz Z, Dubnov J, Friedman-Birnbaum R, Segal I, Zaltzman N, Roth T, Schwartz F, Linn S, Rozenman D, David M, Silbermann M, Barchana M, Bergman R, Sarid R. Infection with Kaposi's sarcoma-associated herpesvirus among families of patients with classic Kaposi's sarcoma. Arch Dermatol. 2005 Nov;141(11):1429-34.
163. Gutzmer R, Al Ghazal M, Geerlings H, Kapp A. Sentinel node biopsy in melanoma delays recurrence but does not change melanoma-related survival: a retrospective analysis of 673 patients. Br J Dermatol. 2005 Dec;153(6):1137-41.
164. Haeck IM, Timmer-de Mik L, Lentjes EG, Buskens E, Hijnen DJ, Guikers C, Bruijnzeel-Koomen CA, de Bruin-Weller MS. Low basal serum cortisol in patients with severe atopic dermatitis: potent topical corticosteroids wrongfully accused. Br J Dermatol. 2007 May;156(5):979-85.
165. Haenssle HA, Korpas B, Hansen-Hagge C, Buhl T, Kaune KM, Johnsen S, Rosenberger A, Schön MP, Emmert S. Selection of patients for long-term surveillance with digital dermoscopy by assessment of melanoma risk factors. Arch Dermatol. 2010 Mar;146(3):257-64.
166. Hald M, Agner T, Blands J, Johansen JD; Danish Contact Dermatitis Group. Delay in medical attention to hand eczema: a follow-up study. Br J Dermatol. 2009 Dec;161(6):1294-300.
167. Hald M, Agner T, Blands J, Veien NK, Laurberg G, Avnstorp C, Menné T, Kaaber K, Kristensen B, Kristensen O, Andersen KE, Paulsen E, Thormann J, Sommerlund M, Nielsen NH, Johansen JD. Clinical severity and prognosis of hand eczema. Br J Dermatol. 2009 Jun;160(6):1229-36.
168. Hald M, Berg ND, Elberling J, Johansen JD. Medical consultations in relation to severity of hand eczema in the general population. Br J Dermatol. 2008 Apr;158(4):773-7.
169. Halevy S, Ghislain PD, Mockenhaupt M, Fagot JP, Bouwes Bavinck JN, Sidoroff A, Naldi L, Dunant A, Viboud C, Roujeau JC; EuroSCAR Study Group. Allopurinol is the most common cause of Stevens-Johnson syndrome and toxic epidermal necrolysis in Europe and Israel. J Am Acad Dermatol. 2008 Jan;58(1):25-32.
170. Halkjaer LB, Loland L, Buchvald FF, Agner T, Skov L, Strand M, Bisgaard H. Development of atopic dermatitis during the first 3 years of life: the Copenhagen prospective study on asthma in childhood cohort study in high-risk children. Arch Dermatol. 2006 May;142(5):561-6.
171. Hall DM, McCarty F, Elliott T, Glanz K. Lifeguards' sun protection habits and sunburns: association with sun-safe environments and skin cancer prevention program participation. Arch Dermatol. 2009 Feb;145(2):139-44.
172. Halpern J, Holder R, Langford NJ. Ethnicity and other risk factors for acute lower limb cellulitis: a U.K.-based prospective case-control study. Br J Dermatol. 2008 Jun;158(6):1288-92.
173. Han J, Qureshi AA, Prescott J, Guo Q, Ye L, Hunter DJ, De Vivo I. A prospective study of telomere length and the risk of skin cancer. J Invest Dermatol. 2009 Feb;129(2):415-21. Epub 2008 Jul 31.
174. Hansen C, Wilkinson D, Hansen M, Argenziano G. How good are skin cancer clinics at melanoma detection? Number needed to treat variability across a national clinic group in Australia. J Am Acad Dermatol. 2009 Oct;61(4):599-604.
175. Harwood CA, Leedham-Green M, Leigh IM, Proby CM. Low-dose retinoids in the prevention of cutaneous squamous cell carcinomas in organ transplant recipients: a 16-year retrospective study. Arch Dermatol. 2005 Apr;141(4):456-64.
176. Harwood CA, Proby CM, McGregor JM, Sheaff MT, Leigh IM, Cerio R. Clinicopathologic features of skin cancer in organ transplant recipients: a retrospective case-control series. J Am Acad Dermatol. 2006 Feb;54(2):290-300.
177. Harwood CA, Surentheran T, Sasieni P, Proby CM, Bordea C, Leigh IM, Wojnarowska F, Breuer J, McGregor JM. Increased risk of skin cancer associated with the presence of epidermodysplasia verruciformis human papillomavirus types in normal skin. Br J Dermatol. 2004 May;150(5):949-57.
178. Hashizume H, Horibe T, Ohshima A, Ito T, Yagi H, Takigawa M. Anxiety accelerates T-helper 2-tilted immune responses in patients with atopic dermatitis. Br J Dermatol. 2005 Jun;152(6):1161-4.
179. Hatta N, Yamada M, Hirano T, Fujimoto A, Morita R. Extramammary Paget's disease: treatment, prognostic factors and outcome in 76 patients. Br J Dermatol. 2008 Feb;158(2):313-8.
180. Hay J, Coups EJ, Ford J, DiBonaventura M. Exposure to mass media health information, skin cancer beliefs, and sun protection behaviors in a United States probability sample. J Am Acad Dermatol. 2009 Nov;61(5):783-92.
181. Hazan C, Dusza SW, Delgado R, Busam KJ, Halpern AC, Nehal KS. Staged excision for lentigo maligna and lentigo maligna melanoma: A retrospective analysis of 117 cases. J Am Acad Dermatol. 2008 Jan;58(1):142-8.
182. Helsing P, Loeb M. Small diameter melanoma: a follow-up of the Norwegian Melanoma Project. Br J Dermatol. 2004 Nov;151(5):1081-3.
183. Herron MD, Hinckley M, Hoffman MS, Papenfuss J, Hansen CB, Callis KP, Krueger GG. Impact of obesity and smoking on psoriasis presentation and management. Arch Dermatol. 2005 Dec;141(12):1527-34.
184. Hervonen K, Vornanen M, Kautiainen H, Collin P, Reunala T. Lymphoma in patients with dermatitis herpetiformis and their first-degree relatives. Br J Dermatol. 2005 Jan;152(1):82-6.
185. Hester EJ, Heilig LF, D'Ambrosia R, Drake AL, Schilling LM, Dellavalle RP. Compliance with youth access regulations for indoor UV tanning. Arch Dermatol. 2005 Aug;141(8):959-62.
186. Heymann AD, Chodick G, Kramer E, Green M, Shalev V. Pemphigus variant associated with penicillin use: a case-cohort study of 363 patients from Israel. Arch Dermatol. 2007 Jun;143(6):704-7.
187. Hicks LD, Cook-Norris RH, Mendoza N, Madkan V, Arora A, Tyring SK. Family history as a risk factor for herpes zoster: a case-control study. Arch Dermatol. 2008 May;144(5):603-8.
188. High WA, Alanen KW, Golitz LE. Is melanocytic nevus with focal atypical epithelioid components (clonal nevus) a superficial variant of deep penetrating nevus? J Am Acad Dermatol. 2006 Sep;55(3):460-6.
189. Hillen U, Jappe U, Frosch PJ, Becker D, Brasch J, Lilie M, Fuchs T, Kreft B, Pirker C, Geier J; German Contact Dermatitis Research Group. Late reactions to the patch-test preparations para-phenylenediamine and epoxy resin: a prospective multicentre investigation of the German Contact Dermatitis Research Group. Br J Dermatol. 2006 Apr;154(4):665-70.
190. Ho SG, Basketter DA, Jefferies D, Rycroft RJ, White IR, McFadden JP. Analysis of para-phenylenediamine allergic patients in relation to strength of patch test reaction. Br J Dermatol. 2005 Aug; 153(2):364-7.
191. Hoerster KD, Mayer JA, Woodruff SI, Malcarne V, Roesch SC, Clapp E. The influence of parents and peers on adolescent indoor tanning behavior: findings from a multi-city sample. J Am Acad Dermatol. 2007 Dec;57(6):990-7.
192. Hsiao JL, Oh DH. The impact of store-and-forward teledermatology on skin cancer diagnosis and treatment. J Am Acad Dermatol. 2008 Aug;59(2):260-7.
193. Hu S, Ma F, Collado-Mesa F, Kirsner RS. UV radiation, latitude, and melanoma in US Hispanics and blacks. Arch Dermatol. 2004 Jul;140(7):819-24.
194. Hu S, Parmet Y, Allen G, Parker DF, Ma F, Rouhani P, Kirsner RS. Disparity in melanoma: a trend analysis of melanoma incidence and stage at diagnosis among whites, Hispanics, and blacks in Florida. Arch Dermatol. 2009 Dec;145(12):1369-74.
195. Hu S, Soza-Vento RM, Parker DF, Kirsner RS. Comparison of stage at diagnosis of melanoma among Hispanic, black, and white patients in Miami-Dade County, Florida. Arch Dermatol. 2006 Jun;142(6):704-8.
196. Hu SC, Chen GS, Wu CS, Chai CY, Chen WT, Lan CC. Rates of cutaneous metastases from different internal malignancies: experience from a Taiwanese medical center. J Am Acad Dermatol. 2009 Mar;60(3):379-87.
197. Huang KP, Weinstock MA, Clarke CA, McMillan A, Hoppe RT, Kim YH. Second lymphomas and other malignant neoplasms in patients with mycosis fungoides and Sezary syndrome: evidence from population-based and clinical cohorts. Arch Dermatol. 2007 Jan;143(1):45-50.
198. Huerta C, Rivero E, Rodríguez LA. Incidence and risk factors for psoriasis in the general population. Arch Dermatol. 2007 Dec;143(12):1559-65.
199. Ismail F, Mitchell L, Casabonne D, Gulati A, Newton R, Proby CM, Harwood CA. Specialist dermatology clinics for organ transplant recipients significantly improve compliance with photoprotection and levels of skin cancer awareness. Br J Dermatol. 2006 Nov;155(5):916-25.
200. Jacobson CC, Resneck JS Jr, Kimball AB. Generational differences in practice patterns of dermatologists in the United States: implications for workforce planning. Arch Dermatol. 2004 Dec;140(12):1477-82.
201. Jagou M, Bastuji-Garin S, Bourdon-Lanoy E, Penso-Assathiany D, Roujeau JC; Réseau d'Epidémiologie en Dermatologie (RED). Poor agreement between self-reported and dermatologists' diagnoses for five common dermatoses. Br J Dermatol. 2006 Nov;155(5):1006-12.
202. Jinnin M, Ihn H, Asano Y, Yamane K, Yazawa N, Tamaki K. Sclerosing panniculitis is associated with pulmonary hypertension in systemic sclerosis. Br J Dermatol. 2005 Sep; 153(3):579-83.
203. Joly P, Benoit-Corven C, Baricault S, Lambert A, Hellot MF, Josset V, Barbaud A, Courville P, Delaporte E, Collet E, Carvalho P, Modeste-Duval AB, Lacour JP, L'Anthoën-Arditi MH, Thuillez C, Benichou J. Chronic eczematous eruptions of the elderly are associated with chronic exposure to calcium channel blockers: results from a case-control study. J Invest Dermatol. 2007 Dec;127(12):2766-71.
204. Jones-Caballero M, Unaeze J, Peñas PF, Stern RS. Use of biological agents in patients with moderate to severe psoriasis: a cohort-based perspective. Arch Dermatol. 2007 Jul;143(7):846-50.
205. Jong CT, Finlay AY, Pearse AD, Kerr AC, Ferguson J, Benton EC, Hawk JL, Sarkany RP, McMullen E, Rhodes LE, Farr PM, Anstey AV. The quality of life of 790 patients with photodermatoses. Br J Dermatol. 2008 Jul;159(1):192-7.
206. Joosse A, Koomen ER, Casparie MK, Herings RM, Guchelaar HJ, Nijsten T. Non-steroidal anti-inflammatory drugs and melanoma risk: large Dutch population-based case-control study. J Invest Dermatol. 2009 Nov;129(11):2620-7.
207. Josefson A, Färm G, Magnuson A, Meding B. Nickel allergy as risk factor for hand eczema: a population-based study. Br J Dermatol. 2009 Apr;160(4):828-34.
208. Jouary T, Legros C, Lalanne N, Versapuech J, Ezzedine K, Vergier B, Delaunay M, Velly JF, Taieb A. Neoadjuvant chemotherapy: a new criterion for selection of candidate patients for surgery of low tumour burden metastases from malignant melanoma? Br J Dermatol. 2010 Jul;163(1):183-7.
209. Jung GW, Metelitsa AI, Dover DC, Salopek TG. Trends in incidence of nonmelanoma skin cancers in Alberta, Canada, 1988-2007. Br J Dermatol. 2010 Jul;163(1):146-54.
210. Kapoor R, Menon C, Hoffstad O, Bilker W, Leclerc P, Margolis DJ. The prevalence of atopic triad in children with physician-confirmed atopic dermatitis. J Am Acad Dermatol. 2008 Jan;58(1):68-73.
211. Karincaoglu Y, Borlu M, Toker SC, Akman A, Onder M, Gunasti S, Usta A, Kandi B, Durusoy C, Seyhan M, Utas S, Saricaoglu H, Ozden MG, Uzun S, Tursen U, Cicek D, Donmez L, Alpsoy E. Demographic and clinical properties of juvenile-onset Behçet's disease: A controlled multicenter study. J Am Acad Dermatol. 2008 Apr;58(4):579-84.
212. Katz KA, Karlawish JH, Chiang DS, Bognet RA, Propert KJ, Margolis DJ. Prevalence and factors associated with use of placebo control groups in randomized controlled trials in psoriasis: a cross-sectional study. J Am Acad Dermatol. 2006 Nov;55(5):814-22.
213. Kaur I, Dogra S, De D, Saikia UN. Histoid leprosy: a retrospective study of 40 cases from India. Br J Dermatol. 2009 Feb;160(2):305-10.
214. Kerr HA, Lim HW. Photodermatoses in African Americans: a retrospective analysis of 135 patients over a 7-year period. J Am Acad Dermatol. 2007 Oct;57(4):638-43.
215. Kho YC, Rhodes LM, Robertson SJ, Su J, Varigos G, Robertson I, Hogan P, Orchard D, Murrell DF. Epidemiology of epidermolysis bullosa in the antipodes the Australasian Epidermolysis Bullosa Registry with a focus on Herlitz junctional epidermolysis bullosa. Arch Dermatol. 2010 Jun;146(6):635-40.
216. Khumalo NP, Jessop S, Gumedze F, Ehrlich R. Hairdressing and the prevalence of scalp disease in African adults. Br J Dermatol. 2007 Nov;157(5):981-8.
217. Kiiski V, de Vries E, Flohil SC, Bijl MJ, Hofman A, Stricker BH, Nijsten T. Risk factors for single and multiple basal cell carcinomas. Arch Dermatol. 2010 Aug;146(8):848-55.
218. Kim CC, Bogart MM, Wee SA, Burstein R, Arndt KA, Dover JS. Predicting migraine responsiveness to botulinum toxin type A injections. Arch Dermatol. 2010 Feb;146(2):159-63.
219. Kimyai-Asadi A, Goldberg LH, Peterson SR, Silapint S, Jih MH. The incidence of major complications from Mohs micrographic surgery performed in office-based and hospital-based settings. J Am Acad Dermatol. 2005 Oct;53(4):628-34.
220. Kinsler VA, Birley J, Atherton DJ. Great Ormond Street Hospital for Children Registry for Congenital Melanocytic Naevi: prospective study 1988-2007. Part 2--Evaluation of treatments. Br J Dermatol. 2009 Feb;160(2):387-92. Epub 2008 Oct 22. Erratum in: Br J Dermatol. 2009 Feb;160(2):474.
221. Kinsler VA, Birley J, Atherton DJ. Great Ormond Street Hospital for Children Registry for congenital melanocytic naevi: prospective study 1988-2007. Part 1-epidemiology, phenotype and outcomes. Br J Dermatol. 2009 Jan;160(1):143-50.
222. Kjellman P, Eriksson H, Berg P. A retrospective analysis of patients with bullous pemphigoid treated with methotrexate. Arch Dermatol. 2008 May;144(5):612-6.
223. Klein RQ, Teal V, Taylor L, Troxel AB, Werth VP. Number, characteristics, and classification of patients with dermatomyositis seen by dermatology and rheumatology departments at a large tertiary medical center. J Am Acad Dermatol. 2007 Dec;57(6):937-43.
224. Krämer U, Lemmen CH, Behrendt H, Link E, Schäfer T, Gostomzyk J, Scherer G, Ring J. The effect of environmental tobacco smoke on eczema and allergic sensitization in children. Br J Dermatol. 2004 Jan;150(1):111-8.
225. Krämer U, Weidinger S, Darsow U, Möhrenschlager M, Ring J, Behrendt H. Seasonality in symptom severity influenced by temperature or grass pollen: results of a panel study in children with eczema. J Invest Dermatol. 2005 Mar;124(3):514-23.
226. Kreuter A, Bischoff S, Skrygan M, Wieland U, Brockmeyer NH, Stücker M, Altmeyer P, Gambichler T. High association of human herpesvirus 8 in large-plaque parapsoriasis and mycosis fungoides. Arch Dermatol. 2008 Aug;144(8):1011-6.
227. Kreuter A, Brockmeyer NH, Weissenborn SJ, Gambichler T, Stücker M, Altmeyer P, Pfister H, Wieland U; German Competence Network HIV/AIDS. Penile intraepithelial neoplasia is frequent in HIV-positive men with anal dysplasia. J Invest Dermatol. 2008 Sep; 128(9):2316-24.
228. Kroft EB, Creemers MC, van den Hoogen FH, Boezeman JB, de Jong EM. Effectiveness, side-effects and period of remission after treatment with methotrexate in localized scleroderma and related sclerotic skin diseases: an inception cohort study. Br J Dermatol. 2009 May;160(5):1075-82.
229. Kroft EB, de Jong EM, Evers AW. Psychological distress in patients with morphea and eosinophilic fasciitis. Arch Dermatol. 2009 Sep;145(9):1017-22.
230. Kulkarni AS, Balkrishnan R, Richmond D, Pearce DJ, Feldman SR. Medication-related factors affecting health care outcomes and costs for patients with psoriasis in the United States. J Am Acad Dermatol. 2005 Jan;52(1):27-31.
231. Kurd SK, Gelfand JM. The prevalence of previously diagnosed and undiagnosed psoriasis in US adults: results from NHANES 2003-2004. J Am Acad Dermatol. 2009 Feb;60(2):218-24.
232. Kurd SK, Troxel AB, Crits-Christoph P, Gelfand JM. The risk of depression, anxiety, and suicidality in patients with psoriasis: a population-based cohort study. Arch Dermatol. 2010 Aug;146(8):891-5.
233. Kütting B, Weistenhöfer W, Baumeister T, Uter W, Drexler H. Current acceptance and implementation of preventive strategies for occupational hand eczema in 1355 metalworkers in Germany. Br J Dermatol. 2009 Aug;161(2):390-6.
234. Kwinter J, Pelletier J, Khambalia A, Pope E. High-potency steroid use in children with vitiligo: a retrospective study. J Am Acad Dermatol. 2007 Feb;56(2):236-41.
235. Durupt F, Mayor L, Bes M, Reverdy ME, Vandenesch F, Thomas L, Etienne J. Prevalence of Staphylococcus aureus toxins and nasal carriage in furuncles and impetigo. Br J Dermatol. 2007 Dec;157(6):1161-7.
236. Lachiewicz AM, Berwick M, Wiggins CL, Thomas NE. Survival differences between patients with scalp or neck melanoma and those with melanoma of other sites in the Surveillance, Epidemiology, and End Results (SEER) program. Arch Dermatol. 2008 Apr;144(4):515-21.
237. Langan SM, Bourke JF, Silcocks P, Williams HC. An exploratory prospective observational study of environmental factors exacerbating atopic eczema in children. Br J Dermatol. 2006 May;154(5):979-80.
238. Langan SM, Hubbard R, Fleming K, West J. A population-based study of acute medical conditions associated with bullous pemphigoid. Br J Dermatol. 2009 Nov;161(5):1149-52.
239. Le Mire L, Hollowood K, Gray D, Bordea C, Wojnarowska F. Melanomas in renal transplant recipients. Br J Dermatol. 2006 Mar;154(3):472-7.
240. LeBlanc WG, Vidal L, Kirsner RS, Lee DJ, Caban-Martinez AJ, McCollister KE, Arheart KL, Chung-Bridges K, Christ S, Clark J 3rd, Lewis JE, Davila EP, Rouhani P, Fleming LE. Reported skin cancer screening of US adult workers. J Am Acad Dermatol. 2008 Jul;59(1):55-63.
241. Lebwohl M, Blum R, Berkowitz E, Kim D, Zitnik R, Osteen C, Wallis WJ. No evidence for increased risk of cutaneous squamous cell carcinoma in patients with rheumatoid arthritis receiving etanercept for up to 5 years. Arch Dermatol. 2005 Jul;141(7):861-4.
242. Lecluse LL, Driessen RJ, Spuls PI, de Jong EM, Stapel SO, van Doorn MB, Bos JD, Wolbink GJ. Extent and clinical consequences of antibody formation against adalimumab in patients with plaque psoriasis. Arch Dermatol. 2010 Feb;146(2):127-32.
243. Lee YL, Li CW, Sung FC, Yu HS, Sheu HM, Guo YL. Environmental factors, parental atopy and atopic eczema in primary-school children: a cross-sectional study in Taiwan. Br J Dermatol. 2007 Dec;157(6):1217-24.
244. Lehman JS, Kalaaji AN. Role of primary prophylaxis for pneumocystis pneumonia in patients treated with systemic corticosteroids or other immunosuppressive agents for immune-mediated dermatologic conditions. J Am Acad Dermatol. 2010 Nov;63(5):815-23.
245. Leibovitch I, Huilgol SC, Selva D, Hill D, Richards S, Paver R. Cutaneous squamous cell carcinoma treated with Mohs micrographic surgery in Australia I. Experience over 10 years. J Am Acad Dermatol. 2005 Aug;53(2):253-60.
246. Leibovitch I, Huilgol SC, Selva D, Paver R, Richards S. Cutaneous lip tumours treated with Mohs micrographic surgery: clinical features and surgical outcome. Br J Dermatol. 2005 Dec;153(6):1147-52.
247. Leibovitch I, Huilgol SC, Selva D, Richards S, Paver R. Basal cell carcinoma treated with Mohs surgery in Australia II. Outcome at 5-year follow-up. J Am Acad Dermatol. 2005 Sep;53(3):452-7.
248. Lembo S, Fallon J, O'Kelly P, Murphy GM. Polymorphic light eruption and skin cancer prevalence: is one protective against the other? Br J Dermatol. 2008 Dec;159(6):1342-7.
249. Lemos BD, Storer BE, Iyer JG, Phillips JL, Bichakjian CK, Fang LC, Johnson TM, Liegeois-Kwon NJ, Otley CC, Paulson KG, Ross MI, Yu SS, Zeitouni NC, Byrd DR, Sondak VK, Gershenwald JE, Sober AJ, Nghiem P. Pathologic nodal evaluation improves prognostic accuracy in Merkel cell carcinoma: analysis of 5823 cases as the basis of the first consensus staging system. J Am Acad Dermatol. 2010 Nov;63(5):751-61.
250. Lewis KG, Nahm WK, Schmidt AN, Moy RL. Lack of difference in the rates of hypopigmentation with 90-microsecond pulsed and longer dwell time carbon-dioxide laser resurfacing. J Am Acad Dermatol. 2004 Feb;50(2):247-52.
251. Lewis KG, Weinstock MA Nonmelanoma skin cancer mortality (1988-2000): the Rhode Island follow-back study Arch Dermatol. 2004 Jul;140(7):837-42
252. Lewis KG, Weinstock MA. Trends in nonmelanoma skin cancer mortality rates in the United States, 1969 through 2000. J Invest Dermatol. 2007 Oct;127(10):2323-7.
253. Li LF, Liu G, Wang J. Prognosis of unclassified eczema: a follow-up study. Arch Dermatol. 2008 Feb;144(2):160-4.
254. Lim JL, Stern RS. High levels of ultraviolet B exposure increase the risk of non-melanoma skin cancer in psoralen and ultraviolet A-treated patients. J Invest Dermatol. 2005 Mar;124(3):505-13.
255. Lindemalm-Lundstam B, Dalenbäck J. Prospective follow-up after curettage-cryosurgery for scalp and face skin cancers. Br J Dermatol. 2009 Sep;161(3):568-76.
256. Linthorst Homan MW, de Korte J, Grootenhuis MA, Bos JD, Sprangers MA, van der Veen JP. Impact of childhood vitiligo on adult life. Br J Dermatol. 2008 Sep;159(4):915-20.
257. Liu W, Dowling JP, Murray WK, McArthur GA, Thompson JF, Wolfe R, Kelly JW. Rate of growth in melanomas: characteristics and associations of rapidly growing melanomas. Arch Dermatol. 2006 Dec;142(12):1551-8.
258. Lovett A, Maari C, Decarie JC, Marcoux D, McCuaig C, Hatami A, Savard P, Powell J. Large congenital melanocytic nevi and neurocutaneous melanocytosis: one pediatric center's experience. J Am Acad Dermatol. 2009 Nov;61(5):766-74.
259. Ludwig RJ, Herzog C, Rostock A, Ochsendorf FR, Zollner TM, Thaci D, Kaufmann R, Vogl TJ, Boehncke WH. Psoriasis: a possible risk factor for development of coronary artery calcification. Br J Dermatol. 2007 Feb;156(2):271-6.
260. Magin P, Sibbritt D, Bailey K. The relationship between psychiatric illnesses and skin disease: a longitudinal analysis of young Australian women. Arch Dermatol. 2009 Aug;145(8):896-902.
261. Makredes M, Robinson D Jr, Bala M, Kimball AB. The burden of autoimmune disease: a comparison of prevalence ratios in patients with psoriatic arthritis and psoriasis. J Am Acad Dermatol. 2009 Sep;61(3):405-10.
262. Malanos D, Stern RS. Psoralen plus ultraviolet A does not increase the risk of cataracts: a 25-year prospective study. J Am Acad Dermatol. 2007 Aug;57(2):231-7.
263. Mälkönen T, Alanko K, Jolanki R, Luukkonen R, Aalto-Korte K, Lauerma A, Susitaival P. Long-term follow-up study of occupational hand eczema. Br J Dermatol. 2010 Nov;163(5):999-1006. doi: 10.1111/j.1365-2133.2010.09987.x.
264. Maragh SL, Brown MD. Prospective evaluation of surgical site infection rate among patients with Mohs micrographic surgery without the use of prophylactic antibiotics. J Am Acad Dermatol. 2008 Aug;59(2):275-8.
265. Marazza G, Pham HC, Schärer L, Pedrazzetti PP, Hunziker T, Trüeb RM, Hohl D, Itin P, Lautenschlager S, Naldi L, Borradori L; Autoimmune bullous disease Swiss study group. Incidence of bullous pemphigoid and pemphigus in Switzerland: a 2-year prospective study. Br J Dermatol. 2009 Oct;161(4):861-8.
266. Margolis DJ, Hoffstad O, Bilker W. Association or lack of association between tetracycline class antibiotics used for acne vulgaris and lupus erythematosus. Br J Dermatol. 2007 Sep;157(3):540-6.
267. Margolis DJ, Hoffstad O, Isseroff RR. Association between the use of beta-adrenergic receptor agents and the development of venous leg ulcers. Arch Dermatol. 2007 Oct;143(10):1275-80.
268. Margolis DJ, Knauss J, Bilker W. Medical conditions associated with venous leg ulcers. Br J Dermatol. 2004 Feb;150(2):267-73.
269. Marie I, Borg JY, Hellot MF, Levesque H. Plasma D-dimer concentration in patients with systemic sclerosis. Br J Dermatol. 2008 Feb;158(2):392-5.
270. Martires KJ, Fu P, Polster AM, Cooper KD, Baron ED. Factors that affect skin aging: a cohort-based survey on twins. Arch Dermatol. 2009 Dec;145(12):1375-9.
271. Marzo-Ortega H, Baxter K, Strauss RM, Drysdale S, Griffiths B, Misbah SA, Gough A, Cunliffe WJ, Emery P. Is minocycline therapy in acne associated with antineutrophil cytoplasmic antibody positivity? A cross-sectional study. Br J Dermatol. 2007 May;156(5):1005-9.
272. Matichard E, Le Hénanff A, Sanders A, Leguyadec J, Crickx B, Descamps V. Effect of neonatal phototherapy on melanocytic nevus count in children. Arch Dermatol. 2006 Dec;142(12):1599-604.
273. Mazereeuw-Hautier J, Syed S, Harper JI. Bilateral facial capillary malformation associated with eye and brain abnormalities. Arch Dermatol. 2006 Aug;142(8):994-8.
274. McBride P, Neale R, Pandeya N, Green A. Sun-related factors, betapapillomavirus, and actinic keratoses: a prospective study. Arch Dermatol. 2007 Jul;143(7):862-8.
275. McCall BP, Horwitz IB, Feldman SR, Balkrishnan R. Incidence rates, costs, severity, and work-related factors of occupational dermatitis: a workers' compensation analysis of Oregon, 1990-1997. Arch Dermatol. 2005 Jun;141(6):713-8.
276. McKenna DB, Lee RJ, Prescott RJ, Doherty VR. A retrospective observational study of primary cutaneous malignant melanoma patients treated with excision only compared with excision biopsy followed by wider local excision. Br J Dermatol. 2004 Mar;150(3):523-30.
277. McKenna DB, Marioni JC, Lee RJ, Prescott RJ, Doherty VR. A comparison of dermatologists', surgeons' and general practitioners' surgical management of cutaneous melanoma. Br J Dermatol. 2004 Sep;151(3):636-44.
278. McPherson T, Persaud S, Singh S, Fay MP, Addiss D, Nutman TB, Hay R. Interdigital lesions and frequency of acute dermatolymphangioadenitis in lymphoedema in a filariasis-endemic area. Br J Dermatol. 2006 May;154(5):933-41.
279. Meding B, Alderling M, Albin M, Brisman J, Wrangsjö K. Does tobacco smoking influence the occurrence of hand eczema? Br J Dermatol. 2009 Mar;160(3):514-8.
280. Meding B, Järvholm B. Incidence of hand eczema-a population-based retrospective study. J Invest Dermatol. 2004 Apr;122(4):873-7.
281. Meding B, Wrangsjö K, Järvholm B. Fifteen-year follow-up of hand eczema: predictive factors. J Invest Dermatol. 2005 May;124(5):893-7.
282. Meding B, Wrangsjö K, Järvholm B. Fifteen-year follow-up of hand eczema: persistence and consequences. Br J Dermatol. 2005 May;152(5):975-80.
283. Meding B, Wrangsjö K, Järvholm B. Hand eczema extent and morphology--association and influence on long-term prognosis. J Invest Dermatol. 2007 Sep;127(9):2147-51.
284. Mehrany K, Weenig RH, Lee KK, Pittelkow MR, Otley CC. Increased metastasis and mortality from cutaneous squamous cell carcinoma in patients with chronic lymphocytic leukemia. J Am Acad Dermatol. 2005 Dec;53(6):1067-71.
285. Mehrany K, Weenig RH, Pittelkow MR, Roenigk RK, Otley CC. High recurrence rates of Basal cell carcinoma after mohs surgery in patients with chronic lymphocytic leukemia. Arch Dermatol. 2004 Aug;140(8):985-8.
286. Metelitsa AI, Dover DC, Smylie M, de Gara CJ, Lauzon GJ. A population-based study of cutaneous melanoma in Alberta, Canada (1993-2002). J Am Acad Dermatol. 2010 Feb;62(2):227-32.
287. Ming ME, Levy R, Hoffstad O, Filip J, Abrams BB, Fernández C, Margolis DJ. The lack of a relationship between atopic dermatitis and nonmelanoma skin cancers. J Am Acad Dermatol. 2004 Mar;50(3):357-62.
288. Miyazaki A, Saida T, Koga H, Oguchi S, Suzuki T, Tsuchida T. Anatomical and histopathological correlates of the dermoscopic patterns seen in melanocytic nevi on the sole: a retrospective study. J Am Acad Dermatol. 2005 Aug;53(2):230-6.
289. Moberg C, Meding B, Stenberg B, Svensson A, Lindberg M. Remembering childhood atopic dermatitis as an adult: factors that influence recollection. Br J Dermatol. 2006 Sep;155(3):557-60.
290. Mockenhaupt M, Viboud C, Dunant A, Naldi L, Halevy S, Bouwes Bavinck JN, Sidoroff A, Schneck J, Roujeau JC, Flahault A. Stevens-Johnson syndrome and toxic epidermal necrolysis: assessment of medication risks with emphasis on recently marketed drugs. The EuroSCAR- study. J Invest Dermatol. 2008 Jan;128(1):35-44.
291. Moehrle M, Kraemer A, Schippert W, Garbe C, Rassner G, Breuninger H. Clinical risk factors and prognostic significance of local recurrence in cutaneous melanoma. Br J Dermatol. 2004 Aug;151(2):397-406.
292. Moffatt CJ, Franks PJ, Doherty DC, Smithdale R, Martin R. Sociodemographic factors in chronic leg ulceration. Br J Dermatol. 2006 Aug;155(2):307-12.
293. Moffatt CJ, Franks PJ, Doherty DC, Smithdale R, Steptoe A. Psychological factors in leg ulceration: a case-control study. Br J Dermatol. 2009 Oct;161(4):750-6.
294. Moghadam-Kia S, Chilek K, Gaines E, Costner M, Rose ME, Okawa J, Werth VP. Cross-sectional analysis of a collaborative Web-based database for lupus erythematosus-associated skin lesions: prospective enrollment of 114 patients. Arch Dermatol. 2009 Mar;145(3):255-60.
295. Mohanna S, Ferrufino JC, Sanchez J, Bravo F, Gotuzzo E. Epidemiological and clinical characteristics of classic Kaposi's sarcoma in Peru. J Am Acad Dermatol. 2005 Sep;53(3):435-41.
296. Möhrenschlager M, Schäfer T, Huss-Marp J, Eberlein-König B, Weidinger S, Ring J, Behrendt H, Krämer U. The course of eczema in children aged 5-7 years and its relation to atopy: differences between boys and girls. Br J Dermatol. 2006 Mar;154(3):505-13.
297. Monestier S, Gaudy C, Gouvernet J, Richard MA, Grob JJ. Multiple senile lentigos of the face, a skin ageing pattern resulting from a life excess of intermittent sun exposure in dark-skinned caucasians: a case-control study. Br J Dermatol. 2006 Mar;154(3):438-44.
298. Moore K, David TJ, Murray CS, Child F, Arkwright PD. Effect of childhood eczema and asthma on parental sleep and well-being: a prospective comparative study. Br J Dermatol. 2006 Mar;154(3):514-8.
299. Morgan MB, Swann M, Somach S, Eng W, Smoller B. Cutaneous angiosarcoma: a case series with prognostic correlation. J Am Acad Dermatol. 2004 Jun;50(6):867-74.
300. Morganroth PA, Kreider ME, Okawa J, Taylor L, Werth VP. Interstitial lung disease in classic and skin-predominant dermatomyositis: a retrospective study with screening recommendations. Arch Dermatol. 2010 Jul;146(7):729-38. Erratum in: Arch Dermatol. 2010 Oct;146(10):1197.
301. Mosher CE, Danoff-Burg S. Addiction to indoor tanning: relation to anxiety, depression, and substance use. Arch Dermatol. 2010 Apr;146(4):412-7.
302. Mourelatos K, Eady EA, Cunliffe WJ, Clark SM, Cove JH. Temporal changes in sebum excretion and propionibacterial colonization in preadolescent children with and without acne. Br J Dermatol. 2007 Jan;156(1):22-31.
303. Mukamal KJ. Alcohol consumption and self-reported sunburn: a cross-sectional, population-based survey. J Am Acad Dermatol. 2006 Oct;55(4):584-9.
304. Murase JE, Chan KK, Garite TJ, Cooper DM, Weinstein GD. Hormonal effect on psoriasis in pregnancy and post partum. Arch Dermatol. 2005 May;141(5):601-6.
305. Naldi L, Chatenoud L, Linder D, Belloni Fortina A, Peserico A, Virgili AR, Bruni PL, Ingordo V, Lo Scocco G, Solaroli C, Schena D, Barba A, Di Landro A, Pezzarossa E, Arcangeli F, Gianni C, Betti R, Carli P, Farris A, Barabino GF, La Vecchia C. Cigarette smoking, body mass index, and stressful life events as risk factors for psoriasis: results from an Italian case-control study. J Invest Dermatol. 2005 Jul;125(1):61-7.
306. Neale RE, Davis M, Pandeya N, Whiteman DC, Green AC. Basal cell carcinoma on the trunk is associated with excessive sun exposure. J Am Acad Dermatol. 2007 Mar;56(3):380-6.
307. Neimann AL, Shin DB, Wang X, Margolis DJ, Troxel AB, Gelfand JM. Prevalence of cardiovascular risk factors in patients with psoriasis. J Am Acad Dermatol. 2006 Nov;55(5):829-35.
308. Nijsten T, Looman CW, Stern RS. Clinical severity of psoriasis in last 20 years of PUVA study. Arch Dermatol. 2007 Sep;143(9):1113-21.
309. Nijsten T, Meads DM, de Korte J, Sampogna F, Gelfand JM, Ongenae K, Evers AW, Augustin M. Cross-cultural inequivalence of dermatology-specific health-related quality of life instruments in psoriasis patients. J Invest Dermatol. 2007 Oct;127(10):2315-22. Epub 2007 May 10.
310. Noe R, Cohen AL, Lederman E, Gould LH, Alsdurf H, Vranken P, Ratard R, Morgan J, Norton SA, Mott J. Skin disorders among construction workers following Hurricane Katrina and Hurricane Rita: an outbreak investigation in New Orleans, Louisiana. Arch Dermatol. 2007 Nov;143(11):1393-8.
311. Novelli M, Merlino C, Ponti R, Bergallo M, Quaglino P, Cambieri I, Comessatti A, Sidoti F, Costa C, Corino D, Cavallo R, Ponzi AN, Fierro MT, Bernengo MG. Epstein-Barr virus in cutaneous T-cell lymphomas: evaluation of the viral presence and significance in skin and peripheral blood. J Invest Dermatol. 2009 Jun;129(6):1556-61.
312. Odenbro A, Gillgren P, Bellocco R, Boffetta P, Håkansson N, Adami J. The risk for cutaneous malignant melanoma, melanoma in situ and intraocular malignant melanoma in relation to tobacco use and body mass index. Br J Dermatol. 2007 Jan;156(1):99-105.
313. Ohtsuka T, Yamazaki S. Increased prevalence of human parvovirus B19 DNA in systemic sclerosis skin. Br J Dermatol. 2004 Jun;150(6):1091-5.
314. Olesen AB, Engholm G, Storm HH, Thestrup-Pedersen K. The risk of cancer among patients previously hospitalized for atopic dermatitis. J Invest Dermatol. 2005 Sep;125(3):445-9.
315. Oliveria SA, Geller AC, Dusza SW, Marghoob AA, Sachs D, Weinstock MA, Buckminster M, Halpern AC. The Framingham school nevus study: a pilot study. Arch Dermatol. 2004 May;140(5):545-51.
316. Olsen AO, Grjibovski A, Magnus P, Tambs K, Harris JR. Psoriasis in Norway as observed in a population-based Norwegian twin panel. Br J Dermatol. 2005 Aug;153(2):346-51.
317. Orringer JS, Helfrich YR, Hamilton T, Friedman C, Johnson TM, Sachs DL. Prevalence of psychotropic medication use among cosmetic and medical dermatology patients: a comparative study. J Am Acad Dermatol. 2006 Mar;54(3):416-9.
318. Pajvani U, Ahmad N, Wiley A, Levy RM, Kundu R, Mancini AJ, Chamlin S, Wagner A, Paller AS. The relationship between family medical history and childhood vitiligo. J Am Acad Dermatol. 2006 Aug;55(2):238-44.
319. Paltiel O, Adler B, Herschko K, Tsukrov B, David M. Are patients with psoriasis susceptible to the classic risk factors for actinic keratoses? Arch Dermatol. 2004 Jul;140(7):805-10.
320. Parker SR, Dyson S, Brisman S, Pennie M, Swerlick RA, Khan R, Manos S, Korman BD, Xia Z, Korman NJ. Mortality of bullous pemphigoid: an evaluation of 223 patients and comparison with the mortality in the general population in the United States. J Am Acad Dermatol. 2008 Oct;59(4):582-8.
321. Patel V, Horn EJ, Lobosco SJ, Fox KM, Stevens SR, Lebwohl M. Psoriasis treatment patterns: results of a cross-sectional survey of dermatologists. J Am Acad Dermatol. 2008 Jun;58(6):964-9.
322. Pelletier F, Bermont L, Puzenat E, Blanc D, Cairey-Remonnay S, Mougin C, Laurent R, Humbert P, Aubin F. Circulating vascular endothelial growth factor in cutaneous malignant melanoma. Br J Dermatol. 2005 Apr;152(4):685-9.
323. Pelucchi C, Di Landro A, Naldi L, La Vecchia C; Oncology Study Group of the Italian Group for Epidemiologic Research in Dermatology (GISED). Risk factors for histological types and anatomic sites of cutaneous basal-cell carcinoma: an italian case-control study. J Invest Dermatol. 2007 Apr;127(4):935-44.
324. Pichon LC, Mayer JA, Hoerster KD, Woodruff SI, Slymen DJ, Belch GE, Clapp EJ, Hurd AL, Forster JL, Weinstock MA. Youth access to artificial UV radiation exposure: practices of 3647 US indoor tanning facilities. Arch Dermatol. 2009 Sep;145(9):997-1002
325. Pink AE, Fonia A, Allen MH, Smith CH, Barker JN. Antinuclear antibodies associate with loss of response to antitumour necrosis factor-alpha therapy in psoriasis: a retrospective, observational study. Br J Dermatol. 2010 Apr;162(4):780-5.
326. Piraccini BM, Sisti A, Tosti A. Long-term follow-up of toenail onychomycosis caused by dermatophytes after successful treatment with systemic antifungal agents. J Am Acad Dermatol. 2010 Mar;62(3):411-4.
327. Placzek M, Arnold B, Schmidt H, Gaube S, Keller E, Plewig G, Degitz K. Elevated 17-hydroxyprogesterone serum values in male patients with acne. J Am Acad Dermatol. 2005 Dec;53(6):955-8.
328. Powell AM, Robson AM, Russell-Jones R, Barlow RJ. Imiquimod and lentigo maligna: a search for prognostic features in a clinicopathological study with long-term follow-up. Br J Dermatol. 2009 May;160(5):994-8.
329. Prinsen CA, Lindeboom R, Sprangers MA, Legierse CM, de Korte J. Health-related quality of life assessment in dermatology: interpretation of Skindex-29 scores using patient-based anchors. J Invest Dermatol. 2010 May;130(5):1318-22.
330. Prinz JC, Kutasi Z, Weisenseel P, Pótó L, Battyáni Z, Ruzicka T. "Borrelia-associated early-onset morphea": a particular type of scleroderma in childhood and adolescence with high titer antinuclear antibodies? Results of a cohort analysis and presentation of three cases. J Am Acad Dermatol. 2009 Feb;60(2):248-55.
331. Prodanovich S, Kirsner RS, Kravetz JD, Ma F, Martinez L, Federman DG. Association of psoriasis with coronary artery, cerebrovascular, and peripheral vascular diseases and mortality. Arch Dermatol. 2009 Jun;145(6):700-3.
332. Prodanovich S, Ma F, Taylor JR, Pezon C, Fasihi T, Kirsner RS. Methotrexat reduces incidence of vascular diseases in veterans with psoriasis or rheumatoid arthritis. J Am Acad Dermatol. 2005 Feb;52(2):262-7**.**
333. Purvis DJ, Thompson JM, Clark PM, Robinson E, Black PN, Wild CJ, Mitchell EA. Risk factors for atopic dermatitis in New Zealand children at 3.5 years of age. Br J Dermatol. 2005 Apr;152(4):742-9.
334. Querfeld C, Rosen ST, Guitart J, Rademaker A, Fung BB, Posten W, Kuzel TM. Comparison of selective retinoic acid receptor- and retinoic X receptor-mediated efficacy, tolerance, and survival in cutaneous t-cell lymphoma. J Am Acad Dermatol. 2004 Jul;51(1):25-32.
335. Querfeld C, Rosen ST, Kuzel TM, Kirby KA, Roenigk HH Jr, Prinz BM, Guitart J. Long-term follow-up of patients with early-stage cutaneous T-cell lymphoma who achieved complete remission with psoralen plus UV-A monotherapy. Arch Dermatol. 2005 Mar;141(3):305-11.
336. Quinn AM, Brown K, Bonish BK, Curry J, Gordon KB, Sinacore J, Gamelli R, Nickoloff BJ. Uncovering histologic criteria with prognostic significance in toxic epidermal necrolysis. Arch Dermatol. 2005 Jun;141(6):683-7.
337. Qureshi AA, Choi HK, Setty AR, Curhan GC. Psoriasis and the risk of diabetes and hypertension: a prospective study of US female nurses. Arch Dermatol. 2009 Apr;145(4):379-82.
338. Raasch BA, Buettner PG, Garbe C. Basal cell carcinoma: histological classification and body-site distribution. Br J Dermatol. 2006 Aug;155(2):401-7.
339. Rajendran PM, Dolev JC, Heaphy MR Jr, Maurer T. Eosinophilic folliculitis: before and after the introduction of antiretroviral therapy. Arch Dermatol. 2005 Oct;141(10):1227-31.
340. Rakkhit T, Panko JM, Christensen TE, Wong B, Nelson T, Papenfuss J, Hansen CB, Callis-Duffin K, Krueger GG. Plaque thickness and morphology in psoriasis vulgaris associated with therapeutic response. Br J Dermatol. 2009 May;160(5):1083-9.
341. Ramachandran S, Rajaratnam R, Smith AG, Lear JT, Strange RC. Patients with both basal and squamous cell carcinomas are at a lower risk of further basal cell carcinomas than patients with only a basal cell carcinoma. J Am Acad Dermatol. 2009 Aug;61(2):247-51.
342. Ramos W, Galarza C, Ronceros G, de Amat F, Teran M, Pichardo L, Juarez D, Anaya R, Mayhua A, Hurtado J, Ortega-Loayza AG. Noninfectious dermatological diseases associated with chronic exposure to mine tailings in a Peruvian district. Br J Dermatol. 2008 Jul;159(1):169-74.
343. Redd JT, Voorhees RE, Török TJ. Outbreak of lepidopterism at a Boy Scout camp. J Am Acad Dermatol. 2007 Jun;56(6):952-5.
344. Rees JR, Stukel TA, Perry AE, Zens MS, Spencer SK, Karagas MR. Tea consumption and basal cell and squamous cell skin cancer: results of a case-control study. J Am Acad Dermatol. 2007 May;56(5):781-5.
345. Regnier S, Fermand V, Levy P, Uzan S, Aractingi S. A case-control study of polymorphic eruption of pregnancy. J Am Acad Dermatol. 2008 Jan;58(1):63-7.
346. Reich K, Krüger K, Mössner R, Augustin M. Epidemiology and clinical pattern of psoriatic arthritis in Germany: a prospective interdisciplinary epidemiological study of 1511 patients with plaque-type psoriasis. Br J Dermatol. 2009 May;160(5):1040-7.
347. Renzi C, Mastroeni S, Passarelli F, Mannooranparampil TJ, Caggiati A, Potenza C, Pasquini P. Factors associated with large cutaneous squamous cell carcinomas. J Am Acad Dermatol. 2010 Sep;63(3):404-11.
348. Revuz JE, Canoui-Poitrine F, Wolkenstein P, Viallette C, Gabison G, Pouget F, Poli F, Faye O, Roujeau JC, Bonnelye G, Grob JJ, Bastuji-Garin S. Prevalence and factors associated with hidradenitis suppurativa: results from two case-control studies. J Am Acad Dermatol. 2008 Oct;59(4):596-601.
349. Ricci G, Patrizi A, Baldi E, Menna G, Tabanelli M, Masi M. Long-term follow-up of atopic dermatitis: retrospective analysis of related risk factors and association with concomitant allergic diseases. J Am Acad Dermatol. 2006 Nov;55(5):765-71.
350. Richards HL, Ling TC, Evangelou G, Brooke RC, Fortune DG, Rhodes LE. Evidence of high levels of anxiety and depression in polymorphic light eruption and their association with clinical and demographic variables. Br J Dermatol. 2008 Aug;159(2):439-44.
351. Richmond-Sinclair NM, Pandeya N, Ware RS, Neale RE, Williams GM, van der Pols JC, Green AC. Incidence of basal cell carcinoma multiplicity and detailed anatomic distribution: longitudinal study of an Australian population. J Invest Dermatol. 2009 Feb;129(2):323-8.
352. Risser J, Lewis K, Weinstock MA. Mortality of bullous skin disorders from 1979 through 2002 in the United States. Arch Dermatol. 2009 Sep;145(9):1005-8.
353. Risser J, Pressley Z, Veledar E, Washington C, Chen SC. The impact of total body photography on biopsy rate in patients from a pigmented lesion clinic. J Am Acad Dermatol. 2007 Sep;57(3):428-34.
354. Rocha-Pereira P, Santos-Silva A, Rebelo I, Figueiredo A, Quintanilha A, Teixeira F. The inflammatory response in mild and in severe psoriasis. Br J Dermatol. 2004 May;150(5):917-28.
355. Rogers HW, Weinstock MA, Harris AR, Hinckley MR, Feldman SR, Fleischer AB, Coldiron BM. Incidence estimate of nonmelanoma skin cancer in the United States, 2006. Arch Dermatol. 2010 Mar;146(3):283-7.
356. Roux O, Lacour JP; Paediatricians of the Region var-Côte d'azur. Eruptive lingual papillitis with household transmission: a prospective clinical study. Br J Dermatol. 2004 Feb;150(2):299-303.
357. Sabel MS, Strecher VJ, Schwartz JL, Wang TS, Karimipour DJ, Orringer JS, Johnson T, Bichakjian CK. Patterns of Internet use and impact on patients with melanoma. J Am Acad Dermatol. 2005 May;52(5):779-85.
358. Salim A, Reece SM, Smith AG, Harrison D, Ramsay HM, Harden PN, Fryer AA.Sebaceous hyperplasia and skin cancer in patients undergoing renal transplant. J Am Acad Dermatol. 2006 Nov;55(5):878-81.
359. Sampogna F, Chren MM, Melchi CF, Pasquini P, Tabolli S, Abeni D; Italian Multipurpose Psoriasis Research on Vital Experiences (Improve) Study Group. Age, gender, quality of life and psychological distress in patients hospitalized with psoriasis. Br J Dermatol. 2006 Feb;154(2):325-31.
360. Sandström MH, Faergemann J. Prognosis and prognostic factors in adult patients with atopic dermatitis: a long-term follow-up questionnaire study. Br J Dermatol. 2004 Jan;150(1):103-10.
361. Saray Y, Seçkin D, Güleç AT, Akgün S, Haberal M. Nail disorders in hemodialysis patients and renal transplant recipients: a case-control study. J Am Acad Dermatol. 2004 Feb;50(2):197-202.
362. Saunes M, Smidesang I, Holmen TL, Johnsen R. Atopic dermatitis in adolescent boys is associated with greater psychological morbidity compared with girls of the same age: the Young-HUNT study. Br J Dermatol. 2007 Feb;156(2):283-8.
363. Schäfer T, Merkl J, Klemm E, Wichmann HE, Ring J; KORA Study Group. The epidemiology of nevi and signs of skin aging in the adult general population: Results of the KORA-survey 2000. J Invest Dermatol. 2006 Jul;126(7):1490-6. Randi G, Naldi L, Gallus S, Di Landro A, La Vecchia C; Oncology Study Group of the Italian Group for Epidemiologic Research in Dermatology (GISED). Number of nevi at a specific anatomical site and its relation to cutaneous malignant melanoma. J Invest Dermatol. 2006 Sep;126(9):2106-10.
364. Scheinfeld N, Mones J. Seasonal variation of transient acantholytic dyskeratosis (Grover's disease). J Am Acad Dermatol. 2006 Aug;55(2):263-8.
365. Schmitt J, Romanos M, Pfennig A, Leopold K, Meurer M. Psychiatric comorbidity in adult eczema. Br J Dermatol. 2009 Oct;161(4):878-83.
366. Schneck J, Fagot JP, Sekula P, Sassolas B, Roujeau JC, Mockenhaupt M. Effects of treatments on the mortality of Stevens-Johnson syndrome and toxic epidermal necrolysis: A retrospective study on patients included in the prospective EuroSCAR Study. J Am Acad Dermatol. 2008 Jan;58(1):33-40.
367. Schneider G, Hockmann J, Ständer S, Luger TA, Heuft G. Psychological factors in prurigo nodularis in comparison with psoriasis vulgaris: results of a case-control study. Br J Dermatol. 2006 Jan;154(1):61-6.
368. Scope A, Marghoob AA, Dusza SW, Satagopan JM, Agero AL, Benvenuto-Andrade C, Lieb JA, Weinstock MA, Oliveria SA, Geller AC, Halpern AC. Dermoscopic patterns of naevi in fifth grade children of the Framingham school system. Br J Dermatol. 2008 May; 158(5):1041-9.
369. Senff NJ, Hoefnagel JJ, Neelis KJ, Vermeer MH, Noordijk EM, Willemze R; Dutch Cutaneous Lymphoma Group. Results of radiotherapy in 153 primary cutaneous B-Cell lymphomas classified according to the WHO-EORTC classification. Arch Dermatol. 2007 Dec;143(12):1520-6.
370. Senff NJ, Willemze R. The applicability and prognostic value of the new TNM classification system for primary cutaneous lymphomas other than mycosis fungoides and Sézary syndrome: results on a large cohort of primary cutaneous B-cell lymphomas and comparison with the system used by the Dutch Cutaneous Lymphoma Group. Br J Dermatol. 2007 Dec;157(6):1205-11.
371. Seoane Reula E, Bellon JM, Gurbindo D, Muñoz-Fernandez MA. Role of antiretroviral therapies in mucocutaneous manifestations in HIV-infected children over a period of two decades. Br J Dermatol. 2005 Aug; 153(2):382-9.
372. Shaikh L, Sagebiel RW, Ferreira CM, Nosrati M, Miller JR 3rd, Kashani-Sabet M. The role of microsatellites as a prognostic factor in primary malignant melanoma. Arch Dermatol. 2005 Jun;141(6):739-42.
373. Shaker OG, El-Tahlawi SM. Is there a relationship between homocysteine and vitiligo? A pilot study. Br J Dermatol. 2008 Sep;159(3):720-4.
374. Shapiro J, Cohen AD, David M, Hodak E, Chodik G, Viner A, Kremer E, Heymann A. The association between psoriasis, diabetes mellitus, and atherosclerosis in Israel: a case-control study. J Am Acad Dermatol. 2007 Apr;56(4):629-34.
375. Shimizu I, Jellinek NJ, Dufresne RG, Li T, Devarajan K, Perlis C. Multiple antithrombotic agents increase the risk of postoperative hemorrhage in dermatologic surgery. J Am Acad Dermatol. 2008 May;58(5):810-6.
376. Shors AR, Kim S, White E, Argenyi Z, Barnhill RL, Duray P, Erickson L, Guitart J, Horenstein MG, Lowe L, Messina J, Rabkin MS, Schmidt B, Shea CR, Trotter MJ, Piepkorn MW. Dysplastic naevi with moderate to severe histological dysplasia: a risk factor for melanoma. Br J Dermatol. 2006 Nov;155(5):988-93.
377. Sidoroff A, Dunant A, Viboud C, Halevy S, Bavinck JN, Naldi L, Mockenhaupt M, Fagot JP, Roujeau JC. Risk factors for acute generalized exanthematous pustulosis (AGEP)-results of a multinational case-control study (EuroSCAR). Br J Dermatol. 2007 Nov;157(5):989-96.
378. Simonart T, De Maertelaer V. Curettage treatment for molluscum contagiosum: a follow-up survey study. Br J Dermatol. 2008 Nov;159(5):1144-7.
379. Skov L, Halkjaer LB, Agner T, Frimodt-Møller N, Jarløv JO, Bisgaard H. Neonatal colonization with Staphylococcus aureus is not associated with development of atopic dermatitis. Br J Dermatol. 2009 Jun;160(6):1286-91.
380. Smith N, Shin DB, Brauer JA, Mao J, Gelfand JM. Use of complementary and alternative medicine among adults with skin disease: results from a national survey. J Am Acad Dermatol. 2009 Mar;60(3):419-25.
381. Socrier Y, Lauwers-Cances V, Lamant L, Garrido I, Lauwers F, Lopez R, Rochaix P, Chevreau C, Payoux P, Viraben R, Paul C, Meyer N. Histological regression in primary melanoma: not a predictor of sentinel lymph node metastasis in a cohort of 397 patients. Br J Dermatol. 2010 Apr;162(4):830-4.
382. Soltani-Arabshahi R, Wong B, Feng BJ, Goldgar DE, Duffin KC, Krueger GG. Obesity in early adulthood as a risk factor for psoriatic arthritis. Arch Dermatol. 2010 Jul;146(7):721-6.
383. Stang A, Jöckel KH. Declining mortality rates for nonmelanoma skin cancers in West Germany, 1968-99. Br J Dermatol. 2004 Mar;150(3):517-22.
384. Stern RS. Lymphoma risk in psoriasis: results of the PUVA follow-up study. Arch Dermatol. 2006 Sep;142(9):1132-5.
385. Stratigos AJ, Malanos D, Touloumi G, Antoniou A, Potouridou I, Polydorou D, Katsambas AD, Whitby D, Mueller N, Stratigos JD, Hatzakis A. Association of clinical progression in classic Kaposi's sarcoma with reduction of peripheral B lymphocytes and partial increase in serum immune activation markers. Arch Dermatol. 2005 Nov;141(11):1421-6.
386. Strauss RM, Elliott F, Affleck P, Boon AP, Newton-Bishop JA. A retrospective study addressed to understanding what predicts severe histological dysplasia/early melanoma in excised atypical melanocytic lesions. Br J Dermatol. 2007 Oct;157(4):758-64.
387. Su LH, Chen TH. Association of androgenetic alopecia with smoking and its prevalence among Asian men: a community-based survey. Arch Dermatol. 2007 Nov;143(11):1401-6.
388. Sun G, Berthelot C, Li Y, Glass DA 2nd, George D, Pandya A, Kurzrock R, Duvic M. Poor prognosis in non-Caucasian patients with early-onset mycosis fungoides. J Am Acad Dermatol. 2009 Feb;60(2):231-5.
389. Synnerstad I, Nilsson L, Fredrikson M, Rosdahl I. Fewer melanocytic nevi found in children with active atopic dermatitis than in children without dermatitis. Arch Dermatol. 2004 Dec;140(12):1471-5.
390. Szepietowski JC, Reich A, Garlowska E, Kulig M, Baran E; Onychomycosis Epidemiology Study Group. Factors influencing coexistence of toenail onychomycosis with tinea pedis and other dermatomycoses: a survey of 2761 patients. Arch Dermatol. 2006 Oct;142(10):1279-84.
391. Tabolli S, Sampogna F, Di Pietro C, Paradisi A, Uras C, Zotti P, Castiglia D, Zambruno G, Abeni D. Quality of life in patients with epidermolysis bullosa. Br J Dermatol. 2009 Oct;161(4):869-77.
392. Taghipour K, Chi CC, Vincent A, Groves RW, Venning V, Wojnarowska F. The association of bullous pemphigoid with cerebrovascular disease and dementia: a case-control study. Arch Dermatol. 2010 Nov;146(11):1251-4.
393. Tan HH, Tan AW, Barkham T, Yan XY, Zhu M. Community-based study of acne vulgaris in adolescents in Singapore. Br J Dermatol. 2007 Sep;157(3):547-51.
394. Tancrède-Bohin E, Ionescu MA, de La Salmonière P, Dupuy A, Rivet J, Rybojad M, Dubertret L, Bachelez H, Lebbé C, Morel P. Prognostic value of blood eosinophilia in primary cutaneous T-cell lymphomas. Arch Dermatol. 2004 Sep;140(9):1057-61.
395. Tang JY, Wu A, Linos E, Parimi N, Lee W, Aszterbaum M, Asgari MM, Bickers DR, Epstein EH Jr. High prevalence of vitamin D deficiency in patients with basal cell nevus syndrome. Arch Dermatol. 2010 Oct;146(10):1105-10.
396. Termorshuizen F, Feltkamp MC, Struijk L, de Gruijl FR, Bavinck JN, van Loveren H. Sunlight exposure and (sero)prevalence of epidermodysplasia verruciformis-associated human papillomavirus. J Invest Dermatol. 2004 Jun;122(6):1456-62.
397. Tessari G, Naldi L, Boschiero L, Nacchia F, Fior F, Forni A, Rugiu C, Faggian G, Sassi F, Gotti E, Fiocchi R, Talamini G, Girolomoni G. Incidence and clinical predictors of a subsequent nonmelanoma skin cancer in solid organ transplant recipients with a first nonmelanoma skin cancer: a multicenter cohort study. Arch Dermatol. 2010 Mar;146(3):294-9.
398. Thieden E, Philipsen PA, Sandby-Møller J, Heydenreich J, Wulf HC. Proportion of lifetime UV dose received by children, teenagers and adults based on time-stamped personal dosimetry. J Invest Dermatol. 2004 Dec;123(6):1147-50.
399. Thieden E, Philipsen PA, Sandby-Møller J, Wulf HC. Sunburn related to UV radiation exposure, age, sex, occupation, and sun bed use based on time-stamped personal dosimetry and sun behavior diaries. Arch Dermatol. 2005 Apr;141(4):482-8.
400. Thieden E, Philipsen PA, Sandby-Møller J, Wulf HC. Sunscreen use related to UV exposure, age, sex, and occupation based on personal dosimeter readings and sun-exposure behavior diaries. Arch Dermatol. 2005 Aug;141(8):967-73.
401. Thyssen JP, Carlsen BC, Menné T, Linneberg A, Nielsen NH, Meldgaard M, Szecsi PB, Stender S, Johansen JD. Filaggrin null mutations increase the risk and persistence of hand eczema in subjects with atopic dermatitis: results from a general population study. Br J Dermatol. 2010 Jul;163(1):115-20.
402. Thyssen JP, Jensen P, Carlsen BC, Engkilde K, Menné T, Johansen JD. The prevalence of chromium allergy in Denmark is currently increasing as a result of leather exposure. Br J Dermatol. 2009 Dec;161(6):1288-93.
403. Thyssen JP, Johansen JD, Carlsen BC, Menné T. Prevalence of nickel and cobalt allergy among female patients with dermatitis before and after Danish government regulation: a 23-year retrospective study. J Am Acad Dermatol. 2009 Nov;61(5):799-805.
404. Thyssen JP, Linneberg A, Menné T, Nielsen NH, Johansen JD. Contact allergy to allergens of the TRUE-test (panels 1 and 2) has decreased modestly in the general population. Br J Dermatol. 2009 Nov;161(5):1124-9.
405. Thyssen JP, Linneberg A, Menné T, Nielsen NH, Johansen JD. The effect of tobacco smoking and alcohol consumption on the prevalence of self-reported hand eczema: a cross-sectional population-based study. Br J Dermatol. 2010 Mar;162(3):619-26.
406. Thyssen JP, Nielsen NH, Linneberg A. The association between alcohol consumption and contact sensitization in Danish adults: the Glostrup Allergy Study. Br J Dermatol. 2008 Feb;158(2):306-12.
407. Titus-Ernstoff L, Perry AE, Spencer SK, Gibson J, Ding J, Cole B, Ernstoff MS. Multiple primary melanoma: two-year results from a population-based study. Arch Dermatol. 2006 Apr;142(4):433-8.
408. Weisshaar E, Schaefer A, Scheidt RR, Bruckner T, Apfelbacher CJ, Diepgen TL. Epidemiology of tick bites and borreliosis in children attending kindergarten or so-called "forest kindergarten" in southwest Germany. J Invest Dermatol. 2006 Mar;126(3):584-90.
409. Tosti A, Bellavista S, Iorizzo M. Alopecia areata: a long term follow-up study of 191 patients. J Am Acad Dermatol. 2006 Sep;55(3):438-41.
410. Tucker SB, Polasek JW, Perri AJ, Goldsmith EA. Long-term follow-up of basal cell carcinomas treated with perilesional interferon alfa 2b as monotherapy. J Am Acad Dermatol. 2006 Jun;54(6):1033-8
411. Tzaneva S, Volc-Platzer B, Kittler H, Hönigsmann H, Tanew A. Antinuclear antibodies in patients with polymorphic light eruption: a long-term follow-up study. Br J Dermatol. 2008 May;158(5):1050-4.
412. Ulrich C, Jürgensen JS, Degen A, Hackethal M, Ulrich M, Patel MJ, Eberle J, Terhorst D, Sterry W, Stockfleth E. Prevention of non-melanoma skin cancer in organ transplant patients by regular use of a sunscreen: a 24 months, prospective, case-control study. BrJ Dermatol. 2009 Nov;161 Suppl 3:78-84.
413. Unaeze J, Nijsten T, Murphy A, Ravichandran C, Stern RS. Impact of psoriasis on health-related quality of life decreases over time: an 11-year prospective study. J Invest Dermatol. 2006 Jul;126(7):1480-9.
414. Valeyrie-Allanore L, Ortonne N, Lantieri L, Ferkal S, Wechsler J, Bagot M, Wolkenstein P. Histopathologically dysplastic neurofibromas in neurofibromatosis 1: diagnostic criteria, prevalence and clinical significance. Br J Dermatol. 2008 May;158(5):1008-12.
415. Valiukeviciene S, Miseviciene I, Gollnick H. The prevalence of common acquired melanocytic nevi and the relationship with skin type characteristics and sun exposure among children in Lithuania. Arch Dermatol. 2005 May;141(5):579-86.
416. van Haalen FM, Bruggink SC, Gussekloo J, Assendelft WJ, Eekhof JA. Warts in primary schoolchildren: prevalence and relation with environmental factors. Br J Dermatol. 2009 Jul;161(1):148-52.
417. Vassallo C, Brazzelli V, Alessandrino PE, Varettoni M, Ardigò M, Lazzarino M, Borroni G. Normal-looking skin in oncohaematological patients after allogeni bone marrow transplantation is not normal. Br J Dermatol. 2004 Sep;151(3):579-86.
418. Vera-Recabarren MA, García-Carrasco M, Ramos-Casals M, Herrero C. Comparative analysis of subacute cutaneous lupus erythematosus and chronic cutaneous lupus erythematosus: clinical and immunological study of 270 patients. Br J Dermatol. 2010 Jan;162(1):91-101.
419. Verhoeven EW, Kraaimaat FW, de Jong EM, Schalkwijk J, van de Kerkhof PC, Evers AW. Individual differences in the effect of daily stressors on psoriasis: a prospective study. Br J Dermatol. 2009 Aug;161(2):295-9.
420. Vestergaard P, Rejnmark L, Mosekilde L. High-dose treatment with vitamin A analogues and risk of fractures. Arch Dermatol. 2010 May;146(5):478-82.
421. Wagamon K, Somach SC, Bass J, Sigel JE, Xue W, Schluchter M, Jaworsky C. Lipidized dermatofibromas and their relationship to serum lipids. J Am Acad Dermatol. 2006 Mar;54(3):494-8.
422. Wahie S, Hiscutt E, Natarajan S, Taylor A. Pityriasis lichenoides: the differences between children and adults. Br J Dermatol. 2007 Nov;157(5):941-5.
423. Wahie S, Lawrence CM. Wound complications following diagnostic skin biopsies in dermatology inpatients. Arch Dermatol. 2007 Oct;143(10):1267-71.
424. Wahl AK, Mørk C, Cooper BA, Padilla G. No long-term changes in psoriasis severity and quality of life following climate therapy. J Am Acad Dermatol. 2005 Apr;52(4):699-701.
425. Wakkee M, Herings RM, Nijsten T. Psoriasis may not be an independent risk factor for acute ischemic heart disease hospitalizations: results of a large population-based Dutch cohort. J Invest Dermatol. 2010 Apr;130(4):962-7.
426. Wakkee M, Lugtenberg M, Spuls PI, de Jong EM, Thio HB, Westert GP, Nijsten T. Knowledge, attitudes and use of the guidelines for the treatment of moderate to severe plaque psoriasis among Dutch dermatologists. Br J Dermatol. 2008 Aug;159(2):426-32.
427. Walker SL, Shah M, Hubbard VG, Pradhan HM, Ghimire M. Skin disease is common in rural Nepal: results of a point prevalence study. Br J Dermatol. 2008 Feb;158(2):334-8.
428. Walling HW. Primary hyperhidrosis increases the risk of cutaneous infection: a case-control study of 387 patients. J Am Acad Dermatol. 2009 Aug;61(2):242-6.
429. Walther U, Kron M, Sander S, Sebastian G, Sander R, Peter RU, Meurer M, Krähn G, Kaskel P. Risk and protective factors for sporadic basal cell carcinoma: results of a two-centre case-control study in southern Germany. Clinical actinic elastosis may be a protective factor. Br J Dermatol. 2004 Jul;151(1):170-8.
430. Wang SQ, Kopf AW, Koenig K, Polsky D, Nudel K, Bart RS. Detection of melanomas in patients followed up with total cutaneous examinations, total cutaneous photography, and dermoscopy. J Am Acad Dermatol. 2004 Jan;50(1):15-20.
431. Warshaw EM, Ahmed RL, Belsito DV, DeLeo VA, Fowler JF Jr, Maibach HI, Marks JG Jr, Toby Mathias CG, Pratt MD, Rietschel RL, Sasseville D, Storrs FJ, Taylor JS, Zug KA; North American Contact Dermatitis Group. Contact dermatitis of the hands: cross-sectional analyses of North American Contact Dermatitis Group Data, 1994-2004. J Am Acad Dermatol. 2007 Aug;57(2):301-14.
432. Warshaw EM, Buchholz HJ, Belsito DV, Maibach HI, Fowler JF Jr, Rietschel RL, Zug KA, Mathias CG, Pratt MD, Sasseville D, Storrs FJ, Taylor JS, Deleo VA, Marks JG Jr. Allergic patch test reactions associated with cosmetics: retrospective analysis of cross-sectional data from the North American Contact Dermatitis Group, 2001-2004. J Am Acad Dermatol. 2009 Jan;60(1):23-38.
433. Warshaw EM, Furda LM, Maibach HI, Rietschel RL, Fowler JF Jr, Belsito DV, Zug KA, DeLeo VA, Marks JG Jr, Mathias CG, Pratt MD, Sasseville D, Storrs FJ, Taylor JS. Anogenital dermatitis in patients referred for patch testing: retrospective analysis of cross-sectional data from the North American Contact Dermatitis Group, 1994-2004. Arch Dermatol. 2008 Jun;144(6):749-55.
434. Waterboer T, Abeni D, Sampogna F, Rother A, Masini C, Sehr P, Michael KM, Pawlita M. Serological association of beta and gamma human papillomaviruses with squamous cell carcinoma of the skin. Br J Dermatol. 2008 Aug;159(2):457-9.
435. Weatherhead SC, Haniffa M, Lawrence CM. Melanomas arising from naevi and de novo melanomas--does origin matter? Br J Dermatol. 2007 Jan;156(1):72-6.
436. Weenig RH, Sewell LD, Davis MD, McCarthy JT, Pittelkow MR. Calciphylaxis: natural history, risk factor analysis, and outcome. J Am Acad Dermatol. 2007 Apr;56(4):569-79.
437. Wenzel J, Brähler S, Bauer R, Bieber T, Tüting T. Efficacy and safety of methotrexate in recalcitrant cutaneous lupus erythematosus: results of a retrospective study in 43 patients. Br J Dermatol. 2005 Jul;153(1):157-62.
438. White JM, McFadden JP, White IR. A review of 241 subjects who were patch tested twice: could fragrance mix I cause active sensitization? Br J Dermatol. 2008 Mar;158(3):518-21.
439. Wieland CN, Comfere NI, Gibson LE, Weaver AL, Krause PK, Murray JA. Anti-bullous pemphigoid 180 and 230 antibodies in a sample of unaffected subjects. Arch Dermatol. 2010 Jan;146(1):21-5.
440. Willemsen R, Vanderlinden J, Roseeuw D, Haentjens P. Increased history of childhood and lifetime traumatic events among adults with alopecia areata. J Am Acad Dermatol. 2009 Mar;60(3):388-93.
441. Wisgerhof HC, Edelbroek JR, de Fijter JW, Feltkamp MC, Willemze R, Bouwes Bavinck JN. Trends of skin diseases in organ-transplant recipients transplanted between 1966 and 2006: a cohort study with follow-up between 1994 and 2006. Br J Dermatol. 2010 Feb 1;162(2):390-6.
442. Wisgerhof HC, van der Boog PJ, de Fijter JW, Wolterbeek R, Haasnoot GW, Claas FH, Willemze R, Bouwes Bavinck JN. Increased risk of squamous-cell carcinoma in simultaneous pancreas kidney transplant recipients compared with kidney transplant recipients. J Invest Dermatol. 2009 Dec;129(12):2886-94
443. Wohl Y, Dreiher J, Cohen AD. Pemphigus and osteoporosis: a case-control study. Arch Dermatol. 2010 Oct;146(10):1126-31.
444. Wolkenstein P, Rodriguez D, Ferkal S, Gravier H, Buret V, Algans N, Simeoni MC, Bastuji-Garin S. Impact of neurofibromatosis 1 upon quality of life in childhood: a cross-sectional study of 79 cases. Br J Dermatol. 2009 Apr;160(4):844-8.
445. Wolkewitz M, Rothenbacher D, Löw M, Stegmaier C, Ziegler H, Radulescu M, Brenner H, Diepgen TL. Lifetime prevalence of self-reported atopic diseases in a population-based sample of elderly subjects: results of the ESTHER study. Br J Dermatol. 2007 Apr;156(4):693-7.
446. Woo DK, Jones CR, Vanoli-Storz MN, Kohler S, Reddy S, Advani R, Hoppe RT, Kim YH. Prognostic factors in primary cutaneous anaplastic large cell lymphoma: characterization of clinical subset with worse outcome. Arch Dermatol. 2009 Jun;145(6):667-74.
447. Xu F, Sheng YY, Mu ZL, Lou W, Zhou J, Ren YT, Qi SS, Wang XS, Fu ZW, Yang QP. Prevalence and types of androgenetic alopecia in Shanghai, China: a community-based study. Br J Dermatol. 2009 Mar;160(3):629-32.
448. Yamamoto Y, Hayashino Y, Yamazaki S, Akiba T, Akizawa T, Asano Y, Saito A, Kurokawa K, Miyachi Y, Fukuhara S; J-DOPPS Research Group. Depressive symptoms predict the future risk of severe pruritus in haemodialysis patients: Japan Dialysis Outcomes and Practice Patterns Study. Br J Dermatol. 2009 Aug;161(2):384-9.
449. Yamamoto Y, Yamazaki S, Hayashino Y, Takahashi O, Tokuda Y, Shimbo T, Fukui T, Hinohara S, Miyachi Y, Fukuhara S. Association between frequency of pruritic symptoms and perceived psychological stress: a Japanese population-based study. Arch Dermatol. 2009 Dec;145(12):1384-8.
450. Yazici C, Köse K, Caliş M, DemIr M, Kirnap M, Ateş F. Increased advanced oxidation protein products in Behçet's disease: a new activity marker? Br J Dermatol. 2004 Jul;151(1):105-11.
451. Zaghloul SS, Cunliffe WJ, Goodfield MJ. Objective assessment of compliance with treatments in acne. Br J Dermatol. 2005 May;152(5):1015-21.
452. Zaghloul SS, Goodfield MJ. Objective assessment of compliance with psoriasis treatment. Arch Dermatol. 2004 Apr;140(4):408-14.
453. Zane LT, Leyden WA, Marqueling AL, Manos MM. A population-based analysis of laboratory abnormalities during isotretinoin therapy for acne vulgaris. Arch Dermatol. 2006 Aug;142(8):1016-22.
454. Zeller S, Lazovich D, Forster J, Widome R. Do adolescent indoor tanners exhibit dependency? J Am Acad Dermatol. 2006 Apr;54(4):589-96.
455. Zug KA, McGinley-Smith D, Warshaw EM, Taylor JS, Rietschel RL, Maibach HI, Belsito DV, Fowler JF Jr, Storrs FJ, DeLeo VA, Marks JG Jr, Mathias CG, Pratt MD, Sasseville D. Contact allergy in children referred for patch testing: North American Contact Dermatitis Group data, 2001-2004. Arch Dermatol. 2008 Oct;144(10):1329-36.
456. Zug KA, Rietschel RL, Warshaw EM, Belsito DV, Taylor JS, Maibach HI, Mathias CG, Fowler JF Jr, Marks JG Jr, DeLeo VA, Pratt MD, Sasseville D, Storrs FJ. The value of patch testing patients with a scattered generalized distribution of dermatitis: retrospective cross-sectional analyses of North American Contact Dermatitis Group data, 2001 to 2004. J Am Acad Dermatol. 2008 Sep;59(3):426-31.
